# The Multilingual CID-5: A New Tool to Study the Perception of Communicative Interactions in Different Languages

**DOI:** 10.3389/fpsyg.2015.01724

**Published:** 2015-11-17

**Authors:** Valeria Manera, Francesco Ianì, Jérémy Bourgeois, Maciej Haman, Łukasz P. Okruszek, Susan M. Rivera, Philippe Robert, Leonhard Schilbach, Emily Sievers, Karl Verfaillie, Kai Vogeley, Tabea von der Lühe, Sam Willems, Cristina Becchio

**Affiliations:** ^1^CoBTeK Laboratory, Faculty of Medicine, University of Nice Sophia AntipolisNice, France; ^2^Department of Psychology, University of TurinTurin, Italy; ^3^Faculty of Psychology, University of WarsawWarsaw, Poland; ^4^Department of Psychology, Center for Mind and Brain & The MIND Institute, University of California, DavisDavis, CA, USA; ^5^Centre Mémoire de Ressources et de Recherche, CHU de NiceNice, France; ^6^Department of Psychiatry, University Hospital CologneCologne, Germany; ^7^Max Planck Institute of PsychiatryMunich, Germany; ^8^Laboratory of Experimental Psychology, KU LeuvenLeuven, Belgium; ^9^Cognitive Neuroscience – Institute of Neuroscience and Medicine (INM3), Research Center JülichJülich, Germany; ^10^Department of Psychiatry and Psychotherapy, Heinrich-Heine-University of Düsseldorf, Rhineland State Clinics DüsseldorfDüsseldorf, Germany; ^11^Department of Robotics, Brain and Cognitive Sciences, Fondazione Istituto Italiano di TecnologiaGenova, Italy

**Keywords:** point-light display, biological motion, communicative interaction, communicative intention, individual intention, cross-linguistic comparisons, forced choice

## Abstract

The investigation of the ability to perceive, recognize, and judge upon social intentions, such as communicative intentions, on the basis of body motion is a growing research area. Cross-cultural differences in ability to perceive and interpret biological motion, however, have been poorly investigated so far. Progress in this domain strongly depends on the availability of suitable stimulus material. In the present method paper, we describe the *multilingual CID-5*, an extension of the CID-5 database, allowing for the investigation of how non-conventional communicative gestures are classified and identified by speakers of different languages. The CID-5 database contains 14 communicative interactions and 7 non-communicative actions performed by couples of agents and presented as point-light displays. For each action, the database provides movie files with the point-light animation, text files with the 3-D spatial coordinates of the point-lights, and five different response alternatives. In the multilingual CID-5 the alternatives were translated into seven languages (Chinese, Dutch, English, French, German, Italian, and Polish). Preliminary data collected to assess the recognizability of the actions in the different languages suggest that, for most of the action stimuli, information presented in point-light displays is sufficient for the distinctive classification of the action as communicative vs. individual, as well as for identification of the specific communicative gesture performed by the actor in all the available languages.

## Introduction

Successful gestural communication depends on the recipient understanding and recognizing the intention of the communicative act (Sperber and Wilson, 1986). To do so, the recipient needs to be able to (a) discriminate between communicative gestures and individual actions (intention classification), and (b) identify the specific communicative content conveyed by the gesture (intention identification). Conventional emblematic communicative gestures, such as the ‘okay’ sign or the ‘thumbs-up’ gesture, have a certain format and an explicit meaning established by the conventions of specific communities. It is thus unsurprising that they may have radically different meanings from one society to another, or even within a single communicative tradition. ‘The horns,’ made by extending the pinkie and index finger while making a fist, for example, is used to ward off the evil eye in traditional Mediterranean cultures. Variants of this gesture were used in Elizabethan England to accuse a man of having an unfaithful wife, in modern England and the US to express a passion for heavy metal music (Casasanto, 2013). Non-conventional gestures, on the contrary, may be more easily understood across cultures. Pointing when giving directions, reaching up to show how tall someone is, gesturing towards an empty seat, are all examples of communicative gestures that can serve as a ‘quasi-universal’ language (Marsh et al., 2007). Comparison of results obtained in different linguistic contexts and cultures, however, have so far been hindered by lack of evaluation instruments adapted and validated in different languages.

In the present work, we describe the *multilingual CID-5*, a new tool being made available in seven languages for the investigation of how non-conventional communicative gestures are classified and identified in different linguistic and cultural contexts. In the following, we present first a brief background on the point-light technique used to create stimuli included in the multilingual CID-5, and in the original CID-5 database. Next, we provide a detailed description of multilingual CID-5 database, including all the materials available for download. Finally, we present normative data collected to assess the stimulus classification (communicative vs. individual) and intention identification by speakers of seven different languages, namely Chinese, Dutch, English, French, German, Italian, and Polish.

### Gestural Communication through Point-light Displays

The point-light technique is a method for representing biological motion through limited visual information (Johansson, 1973). With this method, the movements of a body of a living being are represented by a small number of point lights indicating the major joints of a person performing a given action. Despite the absence of other cues such as contour, color, or texture, observers can quite easily identify what an actor is doing (e.g., Vanrie and Verfaillie, 2004), as well as many features of the actor themselves, including identity (e.g., Loula et al., 2005), gender (e.g., Kozlowski and Cutting, 1977; Pollick et al., 2005; Brooks et al., 2008), age (Montpare and Zebrowitz-McArthur, 1988), emotional state (e.g., Pollick et al., 2001; Atkinson et al., 2004; Clarke et al., 2005), and personality traits (Heberlein et al., 2004).

Given this keen sensitivity to action motion signatures, it is reasonable to expect that people are also able to discern communicative gestures from point-light displays. Along these lines, recent evidence suggests that biological motion information is sufficient for clear classification of a non-conventional action as communicative, as well as for the identification of the specific communicative intent (Manera et al., 2010, 2011a,b, 2013; Centelles et al., 2013). Furthermore, Manera et al. (2011a, 2013) demonstrated that in the context of a communicative interaction between two point-light agents, observing the communicative gesture of one agent enhances the visual discrimination of a second agent who responds appropriately.

The generalizability of these findings across different cultures and linguistic communities, however, is until now poorly documented. There is evidence that biological motion perception is not necessarily influenced by culture, and that point-light stimuli reproducing simple and putatively universal human actions, such as walking, can be recognized even by indigene populations of Amazonian territories (Pica et al., 2011; see also Barrett et al., 2005), as well as by newborns (Simion et al., 2008). It remains possible, however, that cultural tendencies to display particular non-conventional gestures in certain styles influence intention-from-movement judgments, and that speakers of different languages may classify and describe the same actions differently.

### The CID-5 Database

The CID-5 database (Communicative Interaction Database, Five Alternative Forced Choice format, 5AFC) contains 21 full-body point-light stimuli depicting two agents (A and B) engaged either in communicative interactions (*N* = 14) or non-communicative individual actions (*N* = 7) as seen from four different viewpoints. Following Dekeyser et al. (2002), stimuli were constructed by combining motion capture techniques and 3-D animation software to provide precise control over the computer-generated actions and allow the actions of the two agents to be independently manipulated. For each action stimulus, the CID-5 provides (i) coordinate files for each actor; (ii) movie files depicting the action of the two agents as seen from four different perspectives; (iii) five action alternatives describing the action performed by the two agents. The CID-5 database can be freely downloaded from http://bsb-lab.org/research/.

Results collected on a sample of 113 Italian speaking participants using these stimuli confirmed that naive observers are able to distinguish communicative and individual gestures, and to identify the correct action description among the five alternatives (Manera et al., 2015). The *multilingual CID-5* extends the CID-5 by providing a translation of the response alternatives into seven different languages: Chinese, Dutch, English, French, German, Italian, and Polish. Furthermore, it provides some normative data to validate the alternative action descriptions in the different languages.

## The Multilingual CID-5 Database

Building on the CID-5 database, the *multilingual CID-5* database provides a new tool to investigate classification and identification of non-conventional communicative gestures by speakers of different languages. The database is available as Supplementary Material to this article, or from the website of the Biology of Social Behavior Lab, University of Torino (http://bsb-lab.org/research/).

### Actions

A brief description of each action stimulus is reported in **Table 1**. Stimuli consist of the 21 point-light actions depicting two point-light agents, each consisting of 13 markers indicating head, shoulders, elbows, wrists, hips, knees, and feet. For each stimulus, we report the stimulus classification (communicative vs. individual), a brief description of the actions of agent A and agent B, and the actors’ gender.

**Table 1:** Description of the actions included in the CID-5 database.

Action	Action (sequence) description	Communicative vs. individual	Male/female couple
Choose which one	A asks B to choose between two objects; B takes an object.	Communicative	Male
Come closer	A asks B to come closer; B moves forward.	Communicative	Female
Go out of the way	A asks B to go out of the way; B moves over.	Communicative	Male
Imitate me	A squats down and asks B to imitate him; B squats down.	Communicative	Male
Look at the ceiling	A asks B to look at something behind him on the ceiling; B turns around.	Communicative	Male
Look at the ground	A asks B to look at something on the ground; B squats down.	Communicative	Male
Move this down	A asks B to move something down; B picks something and moves it down.	Communicative	Female
No	A says no; B, who had grasped something, puts that down.	Communicative	Male
Pick this up	A points to B something to pick up; B picks something up.	Communicative	Female
Sit down	A asks B to sit down; B sits down.	Communicative	Male
Squat down	A asks B to squat down; B squats down.	Communicative	Female
Stand up	A asks B to stand up; B, who is sitting, stands up.	Communicative	Female
Stop	A asks B to stop; B, who is walking, stops.	Communicative	Male
Walk away	A asks B to walk away; B takes some steps into the indicated direction.	Communicative	Male
Drink	A drinks; B sits down.	Individual	Male
Jump	A jumps; B picks something up.	Individual	Female
Lateral steps	A makes some lateral steps; B takes something and eats it.	Individual	Male
Look under the foot	A looks under his foot; B moves something.	Individual	Female
Sneeze	A sneezes; B turns around.	Individual	Male
Stretch	A stretches; B moves something.	Individual	Female
Turn over	A turns over; B squats down.	Individual	Female

Stimuli were originally constructed by capturing the movements of four actors, two Italian females and two Dutch males, each wearing 30 reflective spherical markers (Qualisys MacReflex motion capture system; Qualisys; Gothenburg, Sweden, consisting of six 30-Hz position units). For the communicative interactions, the two female and the two male actors worked in pair (a couple of male actors and a couple of female actresses) and were assigned to a ‘communicator’ and ‘responder’ role. The communicator (agent A) always initiated the interaction by performing a communicative gesture; the responder (agent B) perceived the communicative gesture and acted in response, based on a predefined interaction plot. To ensure that the responder’s action matched the communicator’s gesture in all respects (e.g., timing, position, kinematics), interactions were captured in real time, with the actors facing each other, at a distance of approximately 2 m. Individual actions were performed by agent A acting in isolation. Objects (e.g., table, chair, coins, fruits) were present during the production of actions to aid the actors in producing natural movements.

After the capture session, the 2-D data from all the position units were processed oﬄine to calculate the 3-D coordinates of the markers. Missing data points (less than 5%) were filled in manually using customized functions of the Fluey 2 motion toolkit (MTK, Televirtual). The data from the markers were then imported into Character Studio (Autodesk Inc, 1998). This allowed to animate a biped for each actor, consisting of a transparent skeleton and 13 bright dots attached to the center of the major joints (shoulders, elbows, wrists, hips, knees, and ankles) and the head. To create the actual movie files, the smoothed data were imported into 3-D Studio as moving bright spheres, and all the frames of the action were rendered as avi-files from four different viewpoints. Some manual smoothing was performed to avoid any remaining “jumpy” dot movements. An orthographic projection was used, and there was no occlusion, so no explicit depth cues were available. To create the communicative action stimuli avi-files, data from the two actors of each couple were imported into the same 3D studio environment, making sure that the actors were exactly at the same distance as in the original recording session. To create the individual action stimuli.avi files, the communicator’s gesture was substituted with an individual action performed by the same actor, making sure to match stimulus duration. Objects present in the scene during motion capturing were never visible in any of the point-light displays.

### Response Alternatives

The ‘Response Alternatives’ folder contains seven.doc files (Supplementary Data Sheet S1) reporting the list of the five response alternatives for each action stimulus in seven different languages (Chinese, Dutch, English, French, German, Italian, and Polish).

The five alternatives included the correct action description and four incorrect response alternatives. The incorrect response alternatives were generated according to the following criteria. For each action stimulus (e.g., A asks B to walk away), two incorrect communicative alternatives (e.g., A opens the door for B; A asks B to move something) and two incorrect non-communicative alternatives (A stretches; A draws a line) were generated by modifying the description of the action of agent A. All alternative action descriptions were constructed to be physically compatible with the action performed by agent A. For instance, if agent A performed an arm movement, then reference to arm movement was included in all incorrect response alternatives describing the action stimulus. Finally, to avoid that for communicative stimuli the correct alternative was selected simply based on the congruence between the actions of the two agents (i.e., agent A asks B to perform an action, and agent B responds *accordingly*), for each action stimulus, one of the incorrect communicative alternatives always described a congruent interaction between the two agents (see Supplementary Table S1). The description of the action of agent B was the same for all response alternatives.

#### Translation of the Alternatives

Translations in each language were performed by two independent native speakers. Translators were provided with the English version of the alternatives, and the original point-light movie files. The two translations were then compared, and in case of discrepancies, the translators were asked to decide together which description matched better the English version of the alternative and the corresponding point-light video.

## Collection of Preliminary Data

### Participants

**FIGURE 1:**
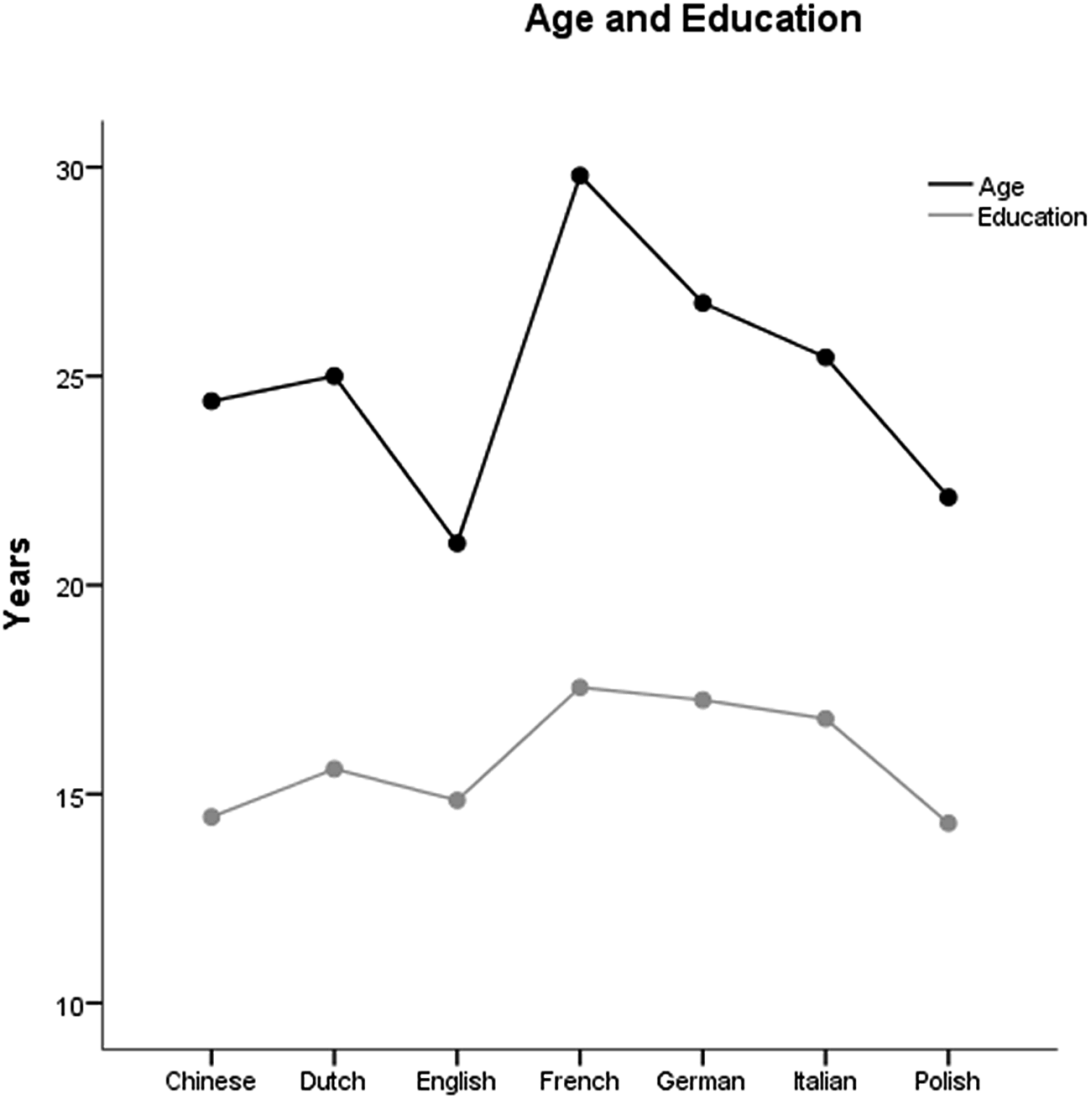
**Mean age and education for the different language groups**.

One hundred and forty healthy volunteers (61 male, 79 female; age, *M* = 24.9, *SD* = 4.6, years of education, *M* = 15.8, *SD* = 2.2) took part in this study, 20 for each of the following languages: Chinese, Dutch, English, French, German, Italian, and Polish. Participants were recruited at the University and Polytechnic of Torino, in Italy (Chinese and Italian speakers), at the Katholieke Universiteit Leuven, in Belgium (Dutch speakers), at the University of California at Davis, in the US (English speakers), at the University Hospital Cologne, in Germany (German speakers), at the University of Nice Sophia Antipolis and the Nice University Hospitals, in France (French speakers), and at the University of Warsaw, in Poland (Polish speakers). They received course credits or payment for their participation. Demographic characteristics of the participants of each country are reported in **Table 2** and **Figure 1**. All participants had normal or corrected to normal vision, and were naive as to the purpose of the study. The study was approved by the local ethical committees.

**Table 2:** Participant’s demographics.

Native Language	Gender *N* of female (in a sample of 20)	Age, years Mean (*SD*)	Education (years) Mean (*SD*)
Chinese	11	24.4 (3.5)	14.5 (1.7)
Dutch	10	25.0 (2.2)	15.6 (1.7)
English	9	21.0 (2.5)	14.9 (1.5)
German	12	26.8 (5.6)	17.3 (1.8)
French	12	29.8 (5.7)	17.6 (2.3)
Italian	10	25.5 (2.6)	16.8 (1.6)
Polish	15	22.1 (2.7)	14.3 (2.1)

### Stimuli and Procedure

Twenty-one point-light actions taken from CID-5 database were employed (Manera et al., 2015), including 14 communicative interactions in which the two agents (A and B) were engaged in a communicative interaction (e.g., agent A points out at the ceiling, agent B looks at the ceiling) and 7 non-communicative individual actions, in which A and B were acting independently of each other (e.g., A drinks, B sits down). Agent A was positioned on the right side of the screen, and agent B was positioned on the left side of the screen (corresponding to the 125° perspective in the CID-5 database; see the description of the video perspectives reported in Manera et al., 2010) in all the action stimuli. The two agents were displayed simultaneously, with action of agent B (the responder in the communicative stimuli) always following in time the action of agent A (the communicator in the communicative stimuli). Stimuli were presented in a randomized order. Following the procedure used in previous reports (e.g., Manera et al., 2015), each video with the two agents was shown twice consecutively, with the two videos separated by a 500 ms fixation cross. After the second presentation of each video, participants were, firstly, asked to decide whether the two agents were communicating vs. acting independently of each other (intention classification). The question was displayed on the screen until a response was provided. Secondly, participants were asked to select the correct action description among five numbered response alternatives displayed simultaneously (intention identification). The order of the response alternatives was randomized across stimuli. The question was presented on the screen until response, with no time restriction. No feedback concerning response correctness was given to the participants. Depending on the sample, instructions, questions, and response alternatives were presented in Chinese, Dutch, English, French, German, Italian, or Polish.

The Chinese and Polish versions of the procedure were created with E-prime software (Psychology Software Tool, Inc), while the Dutch, English, French, German, and Italian versions of the procedure were created with Presentation software (Neurobehavioral Systems, Inc.) In all language samples, stimuli were displayed on a 14″ to 17″ LCD screen. The task took approximately 15–20 min to complete.

### Results

#### Demographics

Chi Square analysis revealed no gender differences among the seven samples corresponding to the different languages (χ^2^ = 4.76, *p* = 0.574). A between-subject ANOVA with age as dependent variable and language (Chinese, Dutch, English, French, German, Italian, and Polish) as between subject factor revealed a significant difference in age among the different language groups [*F*_(6,133)_ = 11.52, *p* < 0.001]. *Post hoc* comparisons (Bonferroni corrected) revealed that French-speaking participants were significantly older than Chinese-speaking participants (*p* < 0.001), Dutch-speaking participants (*p* = 0.003), English-speaking participants (*p* < 0.001), Italian-speaking participants (*p* = 0.010) and Polish-speaking participants (*p* < 0.001). Dutch-speaking participants were significantly older than English-speaking participants (*p* = 0.027). English-speaking participants were younger than German-speaking participants (*p* < 0.001), and Italian-speaking participants (*p* = 0.008). Finally, German-speaking participants were significantly older than Polish-speaking participants (*p* = 0.004).

A between-subject ANOVA with education as dependent variable and language (Chinese, Dutch, English, French, German, Italian, and Polish) as between subject factor revealed a significant difference in education between the different language groups [*F*_(6,133)_= 11.00, *p* < 0.001]. *Post hoc* comparisons (Bonferroni corrected) revealed that French-speaking participants had more education years compared to Chinese-speaking participants (*p* < 0.001), Dutch-speaking participants (*p* = 0.022), English-speaking participants (*p* < 0.001), and Polish-speaking participants (*p* < 0.001). Chinese-speaking participants had fewer education years compared to German-speaking participants (*p* < 0.001). English-speaking participants had fewer education years compared to German-speaking participants (*p* = 0.001) and Italian-speaking participants (*p* = 0.022). Finally, Polish speaking participants had fewer education years compared to German-speaking participants (*p* < 0.001) and Italian-speaking participants (*p* = 0.001).

As age and education differed among the seven language-samples, they were added as covariates in all the between-subject analyses.

#### Multilingual CID-5 Task

Separate analyses were conducted to evaluate global performance and recognizability of single stimuli.

##### Data analysis

*Global performance*. To evaluate global performance, for each language we calculated the percentage of participants who correctly responded to the intention classification and the intention identification questions, and we extracted Signal Detection Theory (SDT) parameters.

To evaluate how participants distinguished between communicative and individual action stimuli (intention classification), we calculated sensitivity (*d*′) and criterion (*c*′). For each participant, we calculated the proportion of hits (arbitrarily defined as “communicative” responses when the action stimulus was communicative) and false alarms (“communicative” responses when the action stimulus was individual). Proportions of 0 were replaced with 0.5/*N*, and proportions of 1 were replaced with (*N*–0.5)/*N* (where *N* is the number of communicative and individual stimuli). *d*′ and *c* were then submitted to single sample *t*-tests (test value = 0) to ascertain whether discrimination performance was above chance level, and to verify the presence of any systematic response bias. Furthermore, to ascertain whether *d*′ and *c* varied across languages, they were submitted to separate ANCOVAs with Language (Chinese, Dutch, English, French, German, Italian, and Polish) and Gender (Male vs. Female) as between-subject factors, and Age and Education as covariates. Finally, in order to verify the presence of interactions between participants’ gender and the gender of the actors in the ability to classify the actions as communicative vs. individual, the *d*′ was submitted to a repeated measures ANOVA with Actor gender as within-subject factor, and Gender as between-subject factor.

To evaluate global performance on the intention identification question, we first recodified responses as communicative vs. individual to calculate sensitivity (*d*′) and criterion (*c*). *d*′ and *c* were submitted to single sample *t*-tests (test value = 0) to ascertain whether discrimination performance was above chance level, and to verify the presence of any systematic response bias. Second, to evaluate the ability to select the correct response alternative, following the standard SDT approach to mAFC (e.g., Macmillan and Creelman, 2005), we used the proportion correct responses as a measure of sensitivity. To compare performance across different languages, we submitted the mean proportion of correct responses to a repeated measures ANCOVA with Intention (Communicative vs. Individual) as within-subject factor, Language (Chinese, Dutch, English, French, German, Italian, and Polish) and Gender (Male vs. Female) as between-subject factors, and Age and Education as covariates. Finally, in order to verify the presence of interactions between participants’ gender and the gender of the actors in the intention identification ability, the proportion of correct responses was submitted to a repeated measures ANOVA with Actor gender as within-subject factor and Gender as between-subject factor.

*Stimulus recognizability*. To provide researchers with detailed data on the classification and identification of single stimuli across languages, for each action stimulus, we first calculated whether the proportion of correct responses differed from chance level – that is, from 0.5 for question 1 (corresponding to 50% of correct responses) and 0.2 for question 2 (corresponding to 20% of correct responses) – by employing binomial tests. Bonferroni corrections were applied to adjust for multiple comparisons (α = 0.05/21, = 0.0023). Second, we verified whether the distribution of correct responses (0 for incorrect response, 1 for correct response) and the distribution of errors (communicative alternative 1, communicative alternative 2, individual alternative 1, and individual alternative 2) varied depending on the factor Language (Chinese, Dutch, English, French, German, Italian, and Polish) by means of Chi-square analyses (see Supplementary Table S1). Bonferroni corrections were applied (*p* < 0.0023).

##### Global performance: results

Descriptive statistics (mean and *SD*) for the percentage of correct responses for the intention classification and the intention identification questions for each language are reported in **Table 3**.

**Table 3:** Percentage of correct responses for the intention classification and identification questions.

Language	Intention classification Mean (*SD*)	Intention identification Mean (*SD*)
Chinese	88% (6%)	71% (7%)
Dutch	90% (6%)	72% (12%)
English	92% (4%)	78% (10%)
German	90% (9%)	80% (9%)
French	90% (8%)	81% (8%)
Italian	89% (8%)	77% (11%)
Polish	89% (9%)	74% (12%)

**FIGURE 2:**
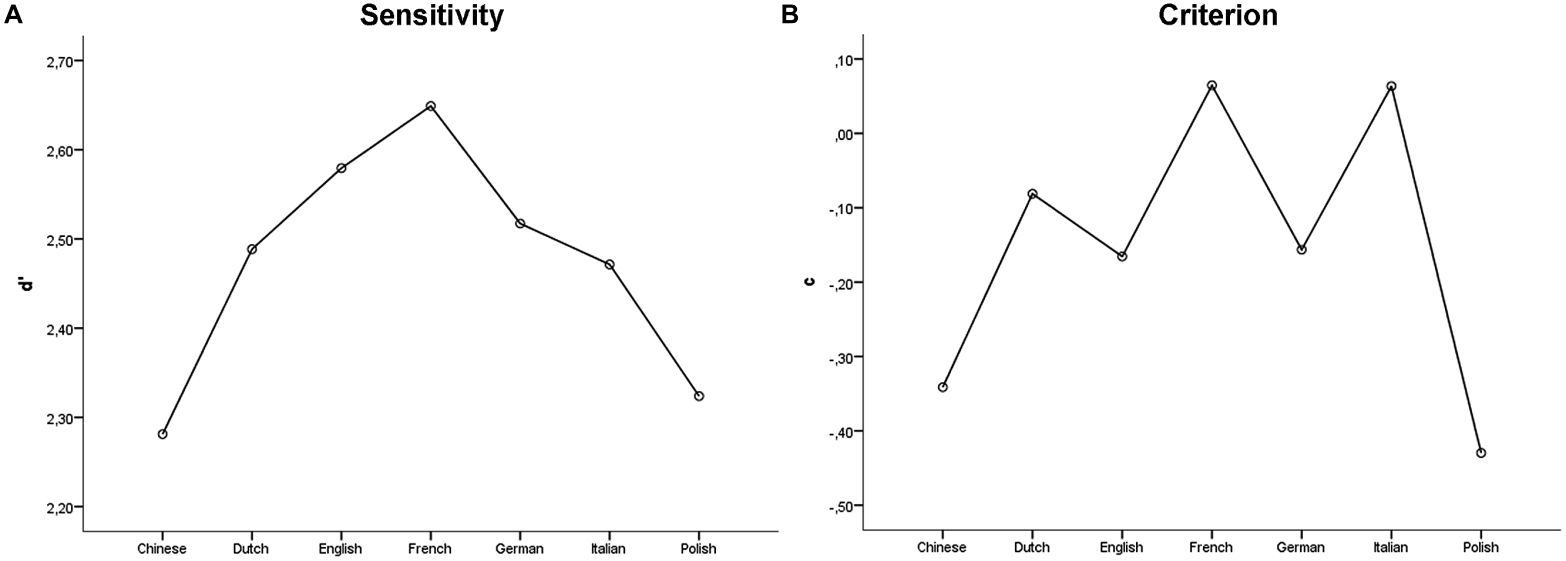
**Sensitivity (A) and criterion (B) for the intention classification question**.

*Intention classification*. Sensitivity (*d*′) for the full sample (*N* = 140) ranged from 0.27 to 3.23 (*M* = 2.46, *SD* = 0.57; see **Figure 2A**), and was significantly higher than zero [*t*_(139)_ = 51.15, *p* < 0.001], thus suggesting that participants, as a group, were able to discriminate communicative from individual action stimuli well above the chance level. The ANCOVA on *d*′ with Language and Gender as between subject factors, and Age and Education as covariates revealed no statistically significant effects [corrected model, *F*_(15,124)_= 0.81, *p* = 0.664]. Specifically, no significant effect of Language [*F*_(6,124)_= 0.83, *p* = 0.546], Gender [*F*_(1,124)_= 0.19, *p* = 0.662], Age [*F*_(1,124)_= 3.48, *p* = 0.064] or Education [*F*_(1,124)_= 0.23, *p* = 0.633] was found. Criterion *c* for the full sample (*N* = 140) ranged from –1.61 to 0.65 (*M* = –0.15, *SD* = 0.36; see **Figure 2B**), and was significantly lower than zero [*t*_(139)_ = –4.84, *p* < 0.001] thus suggesting that participants, as a group, had a tendency to rate stimuli as communicative. The ANCOVA with Language and Gender as between subject factors, and Age and Education as covariates was statistically significant [corrected model, *F*_(15,124)_= 2.95, *p* < 0.001]. Specifically, a main effect of Language was found [*F*_(6,124)_= 4.75, *p* < 0.001]. *Post hoc* comparisons revealed that Chinese-speaking participants had a stronger tendency to rate stimuli as communicative compared to French-speaking (*p* = 0.014) and Italian-speaking participants (*p* = 0.007). Similarly, Polish-speaking participants had a stronger tendency to rate stimuli as communicative compared to French-speaking (*p* = 0.004) and Italian-speaking participants (*p* = 0.001). No significant effect of Gender [*F*_(1,124)_= 3.08, *p* = 0.082], Age [*F*_(1,124)_= 1.54, *p* = 0.218] or Education [*F*_(1,124)_= 0.03, *p* = 0.864] on *c* was found.

The repeated measures ANOVA on *d*′ with Actor gender as within-subject factor and Gender as between-subject factor revealed a significant main effect of Actor Gender [*F*_(1,138)_ = 6.70, *p* = 0.011], with the proportion of correct responses being significantly higher for the female actresses (*M* = 2.56) compared to the male actors (*M* = 2.33). No effect of Gender [*F*_(1,138)_ = 0.92, *p* = 0.340] and no significant interaction between Actor Gender and Gender [*F*_(1,138)_ = 0.06, *p* = 0.815] was found.

*Intention identification*. The *d*′ calculated on the full sample (*N* = 140) after re-codifying the responses as communicative vs. individual ranged from 0.40 to 3.27 (*M* = 2.60, *SD* = 0.53), and was significantly higher than zero [*t*_(139)_ = 58.18, *p* < 0.001], thus suggesting that, also for the action identification question, participants were able to discriminate communicative from individual action stimuli well above the chance level. Criterion *c* calculated on the full sample ranged from –1.27 to 0.65 (*M* = –0.04, *SD* = 0.28), and was not significantly different from zero [*t*_(139)_ = –1.56, *p* = 0.122], thus suggesting that participants, contrary to what happened in the intention classification question, when asked to select the correct action description among several action alternatives, showed no response bias toward a communicative response.

**FIGURE 3:**
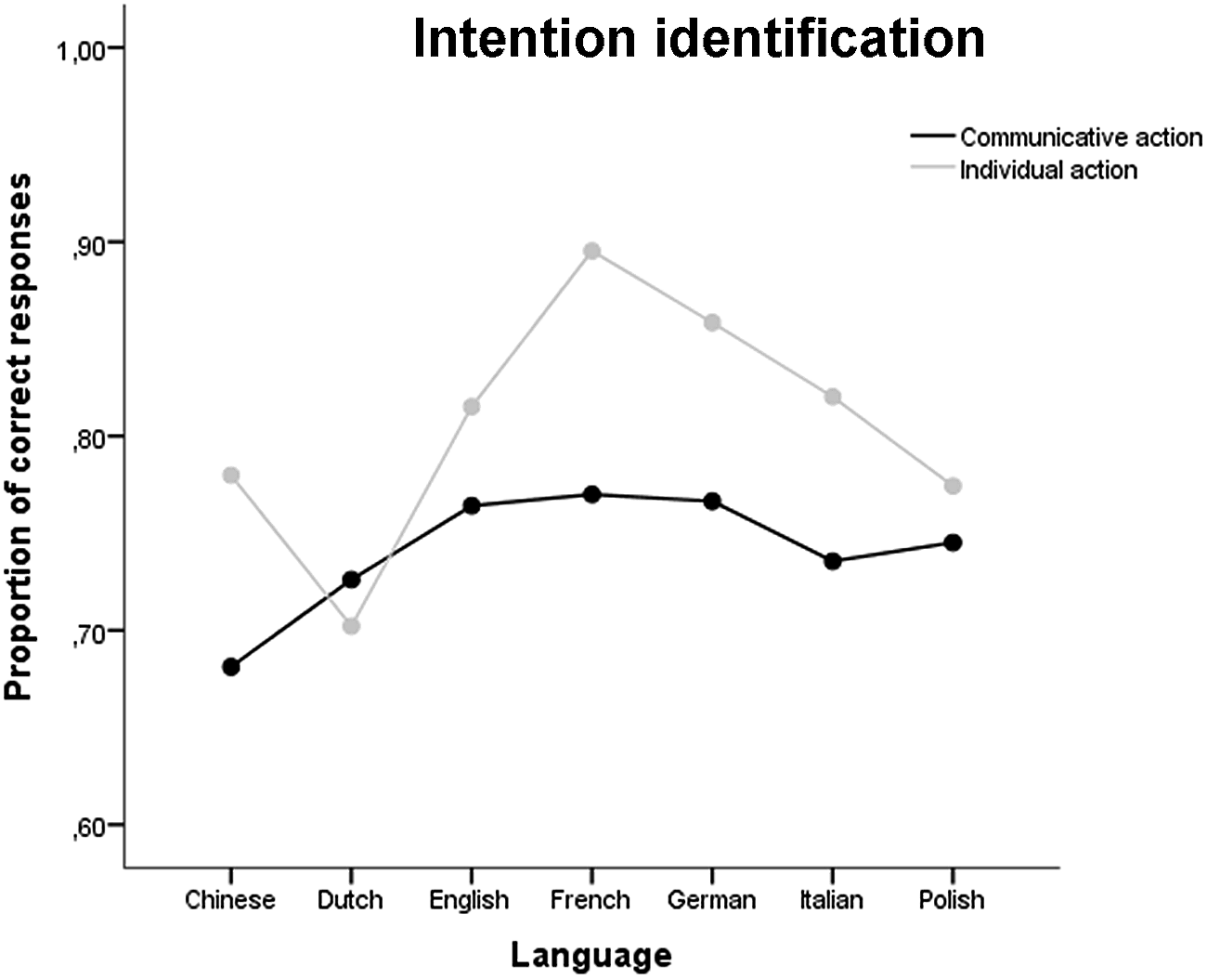
**Proportion of correct responses for the intention identification question**.

The proportion of correct response alternatives for each language is reported in **Figure 3**. The repeated measures ANCOVA with Intention as within-subject factor, Language and Gender as between subject factors and Age and Education as covariates revealed no significant effect of Intention [*F*_(1,124)_= 0.76, *p* = 0.384], Gender [*F*_(1,24)_= 0.27, *p* = 0.606], Age [*F*_(1,124)_= 2.21, *p* = 0.139], or Education [*F*_(1,124)_= 2.13, *p* = 0.147] on the proportion of correct responses. However, a significant effect of Language was found [*F*_(6,124)_= 2.71, *p* = 0.017]. *Post hoc* comparisons revealed that French-speaking participants performed significantly better compared to the Dutch-speaking participants (*p* = 0.026). No two-way or three-way interaction reached statistical significance (all *p*s > 0.056).

The repeated measures ANOVA with Actor gender as within-subject factor and Gender as between-subject factor revealed a significant main effect of Actor Gender [*F*_(1,138)_ = 70.40, *p* < 0001], with the proportion of correct responses being significantly higher for the female actresses (*M* = 0.83) compared to the male actors (*M* = 0.71). No effect of Gender [*F*_(1,138)_ = 0.19, *p* = 0.664] and no significant interaction between Actor Gender and Gender [*F*_(1,138)_ = 1.07, *p* = 0.302] was found.

##### Stimulus recognizability: results

For each stimulus, the percentage of participants who correctly responded to the classification question, and the percentage of participants who reported each of the alternatives in the identification question are reported in Supplementary Table S1.

*Intention classification*. On average, participants correctly classified 90% of the action stimuli as communicative vs. individual (range = 72–99%; S*D* = 8%; Communicative stimuli, *M* = 91%, *SD* = 9%; Individual stimuli, *M* = 87%, *SD* = 8%). The actions that were less consistently recognized were “Look at the ceiling” for the communicative condition (correctly classified as communicative by 74% of the participants) and “Sneeze” for the individual condition (correctly classified as individual by 72% of the participants). Bonferroni corrected binomial tests conducted on the full sample revealed that action classification was above chance level (proportion of correct responses of 0.50) for all the action stimuli (all *p*s < 0.001). Chi-square tests performed on the single action stimuli (intention classification × Language) revealed that intention classification did not differ between languages for any of the 21 actions (see Supplementary Table S1).

*Intention identification*. On average, participants correctly described 76% of the action stimuli (range = 36–96%; *SD* = 17%; Communicative stimuli, *M* = 74%, *SD* = 18%; Individual stimuli, *M* = 80%, *SD* = 14%). Examples of very well recognized stimuli are “Stop” and “Imitate me” for the communicative stimuli, and “Jump” and “Look under the foot” for the individual stimuli. Bonferroni corrected binomial tests conducted on the full sample revealed that action identification was above chance level (proportion of correct responses of 0.20) for all the action stimuli (all *p*s < 0.001). Bonferroni corrected Chi-square tests performed on the single action stimuli (intention identification × Language) revealed that intention identification varied by Language only for the following two actions: “Go out of the way” (*p* < 0.001), and “No” (*p* < 0.001; see Supplementary Table S1). Bonferroni corrected Chi-square tests performed on the errors revealed no significant effect of Language on any of the action stimuli (all *ps* > 0.018), thus suggesting that, when the intention identification was incorrect, participants in the different language samples tended to select the same wrong action alternatives. Some response alternatives were thus more misleading than others in all languages.

## Discussion

In the present paper we describe the *multilingual CID-5*, a database of 21 full-body point-light stimuli depicting two agents engaged in communicative interactions (*N* = 14) or performing non-communicative individual actions (*N* = 7) as seen from different viewpoints. For each stimulus, we provide five plausible response alternatives (only one being correct) translated into seven different languages (Chinese, Dutch, English, French, German, Italian, and Polish). Normative data collected from 140 naive participants (20 participants per language) confirmed that all the stimuli included in the multilingual CID-5 were classified as communicative vs. individual and recognized well above chance level from participants of all the seven language samples. Comparisons of global performance across different languages revealed no difference across samples in the ability to classify actions as communicative vs. individual, as indexed by the SDT parameter *d*′ calculated on the action classification question. Similarly, analyses on the proportion of correct responses divided by action stimulus revealed that all the 21 action stimuli were classified as communicative vs. individual in a comparable way in all language samples, with some actions being consistently very easy (e.g., ‘Stop’) and some others more difficult (e.g., ‘Look at the ceiling’) to classify. Overall, in the intention classification question participants showed a liberal criterion (negative *c*), that is a bias towards reporting the presence of a communicative interaction. This bias may be partially explained by the presence of a greater number of communicative action stimuli. The response bias showed a significant variation across language samples, and was especially evident in the Chinese-speaking and Polish-speaking samples. However, no bias towards reporting a communicative response alternative was found in the intention identification question, when participants were asked to select among multiple response alternatives. Thus, researchers interested in an unbiased measure of the ability to classify stimuli as communicative vs. individual may decide to rely on the intention identification question, after re-coding the response alternatives as communicative vs. individual.

For intention identification (selection of the correct response alternative), we found some individual variations in the proportion of correct responses across language samples, with French-speaking participants performing better compared to Dutch-speaking participants. Analyses divided by action stimulus revealed that 19 out of the 21 stimuli were identified in a comparable way across languages, while only two stimuli (“Go out of the way” and “No”) showed language-dependent variations. Furthermore, the error analysis showed that when the intention identification was incorrect, participants in the different language samples tended to select the same wrong alternative, suggesting that, for most of action stimuli, some response alternatives were more misleading than others in all languages.

These results provide evidence of instrument validity of the multilingual CID-5 as a new tool for the investigation of non-conventional communicative gestures in different languages. It is important to note that our data collection was designed to validate the alternatives in the different languages, and not to explore systematically cultural differences. Thus, from the present results, we cannot conclude that classification and identification of communicative gestures does not vary across cultures. First, participants in the different language samples were not balanced for age and education. Second, Chinese college students were tested in Italy and experienced thus mostly the same environment as Italian participants – a circumstance which might have well influenced their familiarity with the stimulus material. Third, and more importantly, the selected sample groups were not very distant in terms of shared cultural heritage. Future investigations should therefore remain open to the possibilities of systematic difference in nonverbal behavior across distant cultures. A final limitation relates to potential differences in social cognition and visuo-spatial abilities, not assessed in the present study. As individual differences in these abilities have been shown to correlate with recognition of social information from point-light stimuli (Okruszek et al., 2015), taking these variables into account may help to clarify the true nature of cross-linguistic and cross-cultural differences, if any, in intention-from-motion understanding.

## Conflict of Interest Statement

The authors declare that the research was conducted in the absence of any commercial or financial relationships that could be construed as a potential conflict of interest.

## Supplementary material

TABLE S1 **Percentage of participants who correctly responded to the classification question, and who reported each of the alternatives in the identification question.** The “Com vs. Ind” column indicates the percentage of correct responses to the intention classification question (classification of the action as communicative vs. individual). The column “Action” indicates the percentage of responses provided for each of the five response alternatives. The first action alternative (in bold) reports the correct description. The column “Chi Square” reports the Chi Square values calculated on the proportion of correct vs. incorrect responses (intention classification and intention identification by Language). Values indicated in bold are statistically significant (^∗∗∗^*p* < 0.001).
