# Supplementary material for: The Multilingual CID-5: A New Tool to Study the Perception of Communicative Interactions in Different Languages
Source: Front Psychol. 2015 Nov 17;6:1724. doi: 10.3389/fpsyg.2015.01724 (PMC4648072; doi:10.3389/fpsyg.2015.01724)
Supplement: TABLE S1 — Percentage of participants who correctly responded to the classification question, and who reported each of the alternatives in the identification question. The “Com vs. Ind” column indicates the percentage of correct responses to the intention classification question (classification of the action as communicative vs. individual). The column “Action” indicates the percentage of responses provided for each of the five response alternatives. The first action alternative (in bold) reports the correct description. The column “Chi Square” reports the Chi Square values calculated on the proportion of correct vs. incorrect responses (intention classification and intention identification by Language). Values indicated in bold are statistically significant (∗∗∗p < 0.001). [file Table_1.PDF]

Action	Alternative	Alternatives	Chi Square χ^2	Chinese % (N=20)	Dutch % (N=20)	English % (N=20)	French % (N=20)	German % (N=20)	Italian % (N=20)	Polish % (N=20)
Choose which one	**Com vs Ind %**		8.6	**90**	**100**	**100**	**100**	**100**	**95**	**100**
	**Correct (COM)**	**A asks B to choose between two objects. B takes an object**	8.5	**70**	**60**	**50**	**55**	**80**	**55**	**40**
	COM -1	A offers something to B. B takes an object		**5**	20	35	35	20	15	35
	COM -2	A squats down and asks B to imitate him. B takes an object		**0**	0	0	0	0	5	0
	IND -1	A lifts something. B takes an object		**10**	15	5	5	0	15	15
	IND-2	A weights something in his hands. B takes an object		**15**	5	10	5	0	10	10
Come closer	**Com vs Ind %**		6.7	**100**	**100**	**100**	**95**	**90**	**100**	**95**
	**Correct (COM)**	**A asks B to come closer. B moves forward**	17.6	**40**	**75**	**85**	**95**	**70**	**70**	**75**
	COM -1	A shows something to B. B moves forward		**10**	10	0	0	10	0	5
	COM -2	A waves to B. B moves forward		**50**	15	15	0	15	30	20
	IND -1	A drinks. B moves forward		**0**	0	0	5	0	0	0
	IND-2	A stretches. B moves forward		**0**	0	0	0	5	0	0
Go out of the way	**Com vs Ind %**		12.2	**100**	**100**	**100**	**100**	**100**	**100**	**90**
	**Correct (COM)**	**A asks B to go out of the way. B moves over**	**33.2*** **	**20**	**30**	**60**	**90**	**65**	**85**	**55**
	COM -1	A say hello to B. B moves over		**60**	45	20	5	20	0	30
	COM -2	A asks B to hand him something. B moves over		**15**	25	20	0	5	10	5
	IND -1	A scratches himself. B moves over		**5**	0	0	0	10	0	5
	IND-2	A cleans something. B moves over		**0**	0	0	5	0	5	5
Imitate me	**Com vs Ind %**		10.8	**100**	**100**	**95**	**100**	**100**	**85**	**95**
	**Correct (COM)**	**A squats down, and asks B to imitate him. B squats down**	6.3	**100**	**95**	**95**	**95**	**100**	**85**	**95**
	COM -1	A gives something to B. B squats down		**0**	0	0	0	0	5	0
	COM -2	A asks B to come closer. B squats down		**0**	5	0	5	0	0	0
	IND -1	A sits down. B squats down		**0**	0	0	0	0	10	5
	IND-2	A puts something down. B squats down		**0**	0	5	0	0	0	0
Look at the ceiling	**Com vs Ind %**		7.1	**90**	**75**	**70**	**60**	**70**	**65**	**85**
	**Correct (COM)**	**A asks B to look at something behind him on the ceiling. B turns around**	3.0	**45**	**50**	**55**	**35**	**55**	**40**	**40**
	COM -1	A asks B to turn. B turns around		**25**	15	20	10	20	10	30
	COM -2	A asks B to come closer. B turns around		**25**	15	0	25	15	15	20
	IND -1	A drinks. B turns around		**0**	5	25	30	10	25	0

	IND-2	A puts something down. B turns around		**5**	15	0	0	0	10	10
Look at the ground	**Com vs Ind %**		4.1	**95**	**95**	**95**	**100**	**100**	**100**	**100**
	**Correct (COM)**	**A asks B to look at something on the ground. B squats down**	8.1	**90**	**70**	**70**	**65**	**65**	**90**	**65**
	COM -1	A squats down and asks B to imitate him. B squats down		**5**	25	10	30	30	5	35
	COM -2	A asks B to move away. B squats down		**0**	0	0	0	0	0	0
	IND -1	A sits down. B squats down		**5**	5	20	0	5	0	0
	IND-2	A picks something up. B squats down		**0**	0	0	5	0	5	0
Move this down	**Com vs Ind %**		17.9	**85**	**80**	**90**	**55**	**90**	**55**	**90**
	**Correct (COM)**	**A asks B to move something. B moves something**	15.5	**80**	**75**	**90**	**55**	**95**	**55**	**75**
	COM -1	A asks B to imitate him. B moves something		**0**	0	0	0	0	0	5
	COM -2	A asks B to squat down. B moves something		**0**	0	0	0	0	0	0
	IND -1	A pours something. B moves something		**10**	20	5	40	5	40	20
	IND-2	A moves something. B moves something		**10**	5	5	5	0	5	0
No	**Com vs Ind %**		2.1	**95**	**95**	**95**	**100**	**95**	**95**	**100**
	**Correct (COM)**	**A says ‘No’. B stops**	**34.8*** **	**40**	**85**	**90**	**100**	**90**	**95**	**85**
	COM -1	A says ‘Hi’. B stops		**5**	5	10	0	0	0	10
	COM -2	A asks B to get out of the way. B stops		**5**	10	0	0	0	0	0
	IND -1	A rubs something. B stops		**35**	0	0	0	0	5	0
	IND-2	A shakes something. B stops		**15**	0	0	0	10	0	5
Pick this up	**Com vs Ind %**		4.0	**85**	**85**	**95**	**80**	**80**	**85**	**95**
	**Correct (COM)**	**A points to B something to pick up. B picks something up**	5.6	**80**	**90**	**95**	**85**	**75**	**75**	**90**
	COM -1	A asks B to squat down. B picks something up		**10**	0	0	0	5	10	0
	COM -2	A asks B to stop. B picks something up		**0**	0	0	0	0	0	0
	IND -1	A moves something. B picks something up		**0**	0	5	15	0	15	0
	IND-2	A checks the time. B picks something up		**10**	10	0	0	20	0	10
Sit down	**Com vs Ind %**		7.4	**75**	**85**	**90**	**95**	**95**	**80**	**95**
	**Correct (COM)**	**A asks B to sit down. B sits down**	17.3	**70**	**80**	**90**	**95**	**100**	**65**	**95**
	COM -1	A asks B to move over. B sits down		**5**	10	0	0	0	10	0
	COM -2	A gives something to B. B sits down		**0**	0	0	0	0	5	0
	IND -1	A puts something down. B sits down		**15**	5	0	5	0	5	5
	IND-2	A folds something. B sits down		**10**	5	10	0	0	15	0
Squat	**Com vs Ind %**		5.8	**95**	**85**	**95**	**95**	**95**	**85**	**100**
	**Correct (COM)**	**A asks B to squat down. B squats down**	4.0	**85**	**80**	**75**	**95**	**75**	**80**	**85**

down	COM -1	A asks B to pick something up. B squats down		5	5	10	0	15	5	10
	COM -2	A asks B to walk away. B squats down		0	0	0	0	0	0	0
	IND -1	A cleans something. B squats down		0	5	0	5	5	10	0
	IND-2	A bounces a ball. B squats down		10	10	15	0	5	5	5
Stand up	**Com vs Ind %**		4.1	**100**	**95**	**95**	**100**	**95**	**100**	**100**
	**Correct (COM)**	**A asks B to stand up. B stands up**	12.4	**95**	**95**	**80**	**95**	**100**	**95**	**75**
	COM -1	A shows something to B. B stands up		0	5	5	0	0	0	20
	COM -2	A asks B to squat down. B stands up		0	0	0	0	0	0	0
	IND -1	A bounces a ball. B stands up		5	0	5	0	0	0	5
	IND-2	A paints something. B stands up		0	0	10	5	0	5	0
Stop	**Com vs Ind %**		10.8	**100**	**85**	**95**	**95**	**100**	**100**	**100**
	**Correct (COM)**	**A asks B to stop. B stops**	6.3	**100**	**90**	**90**	**100**	**100**	**90**	**95**
	COM -1	A shows something to B. B stops		0	5	5	0	0	10	5
	COM -2	A asks B to sit down. B stops		0	5	0	0	0	0	0
	IND -1	A drinks. B stops		0	0	5	0	0	0	0
	IND-2	A puts something down. B stops		0	0	0	0	0	5	0
Walk away	**Com vs Ind %**		13.5	**95**	**80**	**90**	**60**	**70**	**65**	**90**
	**Correct (COM)**	**A asks B to walk away. B takes some steps**	13.7	**35**	**40**	**30**	**25**	**10**	**55**	**55**
	COM -1	A opens the door for B. B takes some steps		55	35	55	55	70	30	35
	COM -2	A asks B to move something. B takes some steps		5	15	5	5	15	0	0
	IND -1	A stretches. B takes some steps		5	5	10	15	0	5	0
	IND-2	A draws a line. B takes some steps		0	5	0	0	5	10	10
Drink	**Com vs Ind %**		6.6	**90**	**95**	**90**	**100**	**80**	**90**	**80**
	**Correct (IND)**	**A drinks. B sits down**	22.9	**80**	**35**	**85**	**95**	**75**	**80**	**75**
	COM -1	A asks B to sit down. B sits down		5	5	5	0	20	10	10
	COM -2	A asks B to look at something. B sits down		0	15	5	0	5	0	10
	IND -1	A looks at the time. B sits down		10	15	0	0	0	5	0
	IND-2	A scratches his head. B sits down		5	30	5	5	0	5	5
Jump	**Com vs Ind %**		7.8	**100**	**100**	**100**	**100**	**95**	**90**	**90**
	**Correct (IND)**	**A jumps. B picks something up**	10.8	**100**	**100**	**100**	**95**	**95**	**100**	**85**
	COM -1	A tells B he is very happy. B picks something up		0	0	0	0	0	0	5
	COM -2	A asks B to pick something up. B picks something up		0	0	0	0	0	0	10
	IND -1	A moves over. B picks something up		0	0	0	0	5	0	0
	IND-2	A lifts something. B picks something up		0	0	0	5	0	0	0

Lateral steps	**Com vs Ind %**		2.8	**75**	**90**	**85**	**90**	**85**	**90**	**85**
	**Correct (IND)**	**A makes some lateral steps. B takes something and eats it**	8.4	**65**	**80**	**85**	**80**	**80**	**55**	**60**
	COM -1	A offers something to B. B takes something and tastes it		10	5	15	0	10	15	25
	COM -2	A asks B to move over. B takes something and eats it		5	5	0	5	0	5	0
	IND -1	A turns around. B takes something and eats it		0	5	0	0	5	15	0
	IND-2	A pushes something. B takes something and eats it		20	5	0	15	5	10	15
Look under the foot	**Com vs Ind %**		11.1	**80**	**95**	**100**	**95**	**90**	**100**	**80**
	**Correct (IND)**	**A looks under his foot. B moves something**	8.4	**85**	**100**	**95**	**95**	**100**	**100**	**90**
	COM -1	A asks B to move something. B moves something		5	0	0	0	0	0	0
	COM -2	A asks B to look at something. B moves something		5	0	0	0	0	0	5
	IND -1	A kicks something. B moves something		5	0	5	0	0	0	5
	IND-2	A turns around. B moves something		0	0	0	5	0	0	0
Sneeze	**Com vs Ind %**		16.3	**55**	**75**	**70**	**75**	**80**	**100**	**50**
	**Correct (IND)**	**A sneezes. B turns around**	8.3	**70**	**55**	**85**	**85**	**80**	**85**	**75**
	COM -1	A asks B to look at something. B turns around		5	5	0	0	5	0	15
	COM -2	A asks B to sit down. B turns around		5	15	0	5	5	5	5
	IND -1	A eats something. B turns around		15	5	5	0	0	0	0
	IND-2	A throws something on the ground. B turns around		5	20	10	10	10	10	5
Stretch	**Com vs Ind %**		14.0	**70**	**70**	**90**	**95**	**85**	**100**	**70**
	**Correct (IND)**	**A stretches. B moves something**	19.1	**40**	**30**	**60**	**65**	**75**	**85**	**45**
	COM -1	A asks B to move something. B moves something		5	25	15	10	0	0	5
	COM -2	A asks B to squat down. B moves something		0	5	0	0	5	0	0
	IND -1	A picks something up. B moves something		45	35	25	25	15	15	40
	IND-2	A moves something. B moves something		10	5	0	0	5	0	10
Turn over	**Com vs Ind %**		13.7	**80**	**100**	**100**	**100**	**95**	**85**	**80**
	**Correct (IND)**	**A turns over. B squats down**	14.0	**95**	**90**	**75**	**100**	**100**	**75**	**90**
	COM -1	A shows something to B. squats down		0	5	0	0	0	5	5
	COM -2	A asks B to move away. B squats down		0	0	0	0	0	0	5
	IND -1	A moves something. B squats down		0	0	0	0	0	5	0
	IND-2	A moves away. B squats down		5	5	25	0	0	15	0
