# Supplementary material for: The Multilingual CID-5: A New Tool to Study the Perception of Communicative Interactions in Different Languages
Source: Front Psychol. 2015 Nov 17;6:1724. doi: 10.3389/fpsyg.2015.01724 (PMC4648072; doi:10.3389/fpsyg.2015.01724)
Supplement: Supplementary file 2 [file Data_Sheet_1.ZIP › Alternatives/chinese.doc]

| **Action** | **Alternative** | **Alternatives** | **Chinese**  **% (N=20)** |  |
| --- | --- | --- | --- | --- |
| Choose which one | **Com vs Ind %** |  | **90** |  |
|  | **Correct (COM)** | **A asks B to choose between two objects. B takes an object** | **70** | **A让B从两个物品中选一个，B拿了一个物品。** |
|  | COM -1 | A offers something to B. B takes an object | **5** | **A给B某物，B拿了一个物品。** |
|  | COM -2 | A squats down and asks B to imitate him. B takes an object | **0** | **A蹲下并让B学他，B拿了一个物品。** |
|  | IND -1 | A lifts something. B takes an object | **10** | **A抬起某物，B拿了一个物品。** |
|  | IND-2 | A weights something in his hands. B takes an object | **15** | **A掂了下手里的物品，B拿了一个物品。** |
| Come closer | **Com vs Ind %** |  | **100** |  |
|  | **Correct (COM)** | **A asks B to come closer. B moves forward** | **40** | **A 让 B靠近，B向前移动。** |
|  | COM -1 | A shows something to B. B moves forward | **10** | **A给B看某物，B向前移动。** |
|  | COM -2 | A waves to B. B moves forward | **50** | **A向B招手，B向前移动。** |
|  | IND -1 | A drinks. B moves forward | **0** | **A喝水，B向前移动。** |
|  | IND-2 | A stretches. B moves forward | **0** | **A伸展，B向前移动。** |
| Go out of the way | **Com vs Ind %** |  | **100** |  |
|  | **Correct (COM)** | **A asks B to go out of the way. B moves over** | **20** | **A让B让出道来，B走了过来。** |
|  | COM -1 | A say hello to B. B moves over | **60** | **A和B打招呼，B走了过来。** |
|  | COM -2 | A asks B to hand him something. B moves over | **15** | **A让B递给他某物，B走了过来。** |
|  | IND -1 | A scratches himself. B moves over | **5** | **A挠痒，B走了过来。** |
|  | IND-2 | A cleans something. B moves over | **0** | **A清理某物，B走了过来。** |
| Imitate me | **Com vs Ind %** |  | **100** |  |
|  | **Correct (COM)** | **A squats down, and asks B to imitate him. B squats down** | **100** | **A蹲下并让B学他，B蹲下。** |
|  | COM -1 | A gives something to B. B squats down | **0** | **A给B某物，B蹲下。** |
|  | COM -2 | A asks B to come closer. B squats down | **0** | **A让B靠近，B蹲下。** |
|  | IND -1 | A sits down. B squats down | **0** | **A坐下，B蹲下。** |
|  | IND-2 | A puts something down. B squats down | **0** | **A放下某物，B蹲下。** |
| Look at the ceiling | **Com vs Ind %** |  | **90** |  |
|  | **Correct (COM)** | **A asks B to look at something behind him on the ceiling. B turns around** | **45** | **A让B去看B身后天花板上的某物，B转身。** |
|  | COM -1 | A asks B to turn. B turns around | **25** | **A让B转身，B转身。** |
|  | COM -2 | A asks B to come closer. B turns around | **25** | **A让B靠近，B转身。** |
|  | IND -1 | A drinks. B turns around | **0** | **A喝水，B转身。** |
|  | IND-2 | A puts something down. B turns around | **5** | **A放下某物，B转身。** |
| Look at the ground | **Com vs Ind %** |  | **95** |  |
|  | **Correct (COM)** | **A asks B to look at something on the ground. B squats down** | **90** | **A让B看地上的某物，B蹲下。** |
|  | COM -1 | A squats down and asks B to imitate him. B squats down | **5** | **A蹲下并让B学他，B蹲下。** |
|  | COM -2 | A asks B to move away. B squats down | **0** | **A让B走开，B蹲下。** |
|  | IND -1 | A sits down. B squats down | **5** | **A坐下，B蹲下。** |
|  | IND-2 | A picks something up. B squats down | **0** | **A捡起某物，B蹲下。** |
| Move this down | **Com vs Ind %** |  | **85** |  |
|  | **Correct (COM)** | **A asks B to move something. B moves something** | **80** | **A让B移动某物，B移动某物。** |
|  | COM -1 | A asks B to imitate him. B moves something | **0** | **A让B学他，B移动某物。** |
|  | COM -2 | A asks B to squat down. B moves something | **0** | **A让B蹲下，B移动某物。** |
|  | IND -1 | A pours something. B moves something | **10** | **A倾倒某物，B移动某物。** |
|  | IND-2 | A moves something. B moves something | **10** | **A移动某物，B移动某物。** |
| No | **Com vs Ind %** |  | **95** |  |
|  | **Correct (COM)** | **A says ‘No’. B stops** | **40** | **A说：“不”，B停下。** |
|  | COM -1 | A says ‘Hi’. B stops | **5** | **A说：“你好”，B停下。** |
|  | COM -2 | A asks B to get out of the way. B stops | **5** | **A让B让出道来，B停下。** |
|  | IND -1 | A rubs something. B stops | **35** | **A摩擦某物，B停下。** |
|  | IND-2 | A shakes something. B stops | **15** | **A摇晃某物，B停下。** |
| Pick this up | **Com vs Ind %** |  | **85** |  |
|  | **Correct (COM)** | **A points to B something to pick up. B picks something up** | **80** | **A让B捡起某物，B捡起某物。** |
|  | COM -1 | A asks B to squat down. B picks something up | **10** | **A让B蹲下，B捡起某物。** |
|  | COM -2 | A asks B to stop. B picks something up | **0** | **A让B停止，B捡起某物。** |
|  | IND -1 | A moves something. B picks something up | **0** | **A移动某物，B捡起某物。** |
|  | IND-2 | A checks the time. B picks something up | **10** | **A查看时间，B捡起某物。** |
| Sit down | **Com vs Ind %** |  | **75** |  |
|  | **Correct (COM)** | **A asks B to sit down. B sits down** | **70** | **A让B坐下，B坐下。** |
|  | COM -1 | A asks B to move over. B sits down | **5** | **A让B走开，B坐下。** |
|  | COM -2 | A gives something to B. B sits down | **0** | **A给B某物，B坐下。** |
|  | IND -1 | A puts something down. B sits down | **15** | **A放下某物，B坐下。** |
|  | IND-2 | A folds something. B sits down | **10** | **A折叠某物，B坐下。** |
| Squat down | **Com vs Ind %** |  | **95** |  |
|  | **Correct (COM)** | **A asks B to squat down. B squats down** | **85** | **A让B蹲下，B蹲下。** |
|  | COM -1 | A asks B to pick something up. B squats down | **5** | **A让B捡起某物，B蹲下。** |
|  | COM -2 | A asks B to walk away. B squats down | **0** | **A让B走开，B蹲下。** |
|  | IND -1 | A cleans something. B squats down | **0** | **A清理某物，B蹲下。** |
|  | IND-2 | A bounces a ball. B squats down | **10** | **A拍球，B蹲下。** |
| Stand up | **Com vs Ind %** |  | **100** |  |
|  | **Correct (COM)** | **A asks B to stand up. B stands up** | **95** | **A让B站起来，B站起来。** |
|  | COM -1 | A shows something to B. B stands up | **0** | **A给B看某物，B站起来。** |
|  | COM -2 | A asks B to squat down. B stands up | **0** | **A让B蹲下，B站起来。** |
|  | IND -1 | A bounces a ball. B stands up | **5** | **A拍球，B站起来。** |
|  | IND-2 | A paints something. B stands up | **0** | **A画某物，B站起来。** |
| Stop | **Com vs Ind %** |  | **100** |  |
|  | **Correct (COM)** | **A asks B to stop. B stops** | **100** | **A让B停止，B停止。** |
|  | COM -1 | A shows something to B. B stops | **0** | **A给B看某物，B停止。** |
|  | COM -2 | A asks B to sit down. B stops | **0** | **A让B坐下，B停止。** |
|  | IND -1 | A drinks. B stops | **0** | **A喝水，B停止。** |
|  | IND-2 | A puts something down. B stops | **0** | **A放下某物，B停止。** |
| Walk away | **Com vs Ind %** |  | **95** |  |
|  | **Correct (COM)** | **A asks B to walk away. B takes some steps** | **35** | **A让B走开，B走了几步。** |
|  | COM -1 | A opens the door for B. B takes some steps | **55** | **A为B开门，B走了几步。** |
|  | COM -2 | A asks B to move something. B takes some steps | **5** | **A让B移动某物，B走了几步。** |
|  | IND -1 | A stretches. B takes some steps | **5** | **A伸展，B走了几步。** |
|  | IND-2 | A draws a line. B takes some steps | **0** | **A画了条线，B走了几步。** |
| Drink | **Com vs Ind %** |  | **90** |  |
|  | **Correct (IND)** | **A drinks. B sits down** | **80** | **A让B坐下，B坐下。** |
|  | COM -1 | A asks B to sit down. B sits down | 5 | **A让B看某个东西，B坐下。** |
|  | COM -2 | A asks B to look at something. B sits down | 0 | **A喝水，B坐下。** |
|  | IND -1 | A looks at the time. B sits down | 10 | **A看时间，B坐下。** |
|  | IND-2 | A scratches his head. B sits down | 5 | **A挠头，B坐下。** |
| Jump | **Com vs Ind %** |  | **100** |  |
|  | **Correct (IND)** | **A jumps. B picks something up** | **100** | **A告诉B他很快乐，B捡起某物。** |
|  | COM -1 | A tells B he is very happy. B picks something up | 0 | **A让B捡起某物，B捡起某物。** |
|  | COM -2 | A asks B to pick something up. B picks something up | 0 | **A跳跃，B捡起某物。** |
|  | IND -1 | A moves over. B picks something up | 0 | **A走过来，B捡起某物。** |
|  | IND-2 | A lifts something. B picks something up | 0 | **A提起某物，B捡起某物。** |
| Lateral steps | **Com vs Ind %** |  | **75** |  |
|  | **Correct (IND)** | **A makes some lateral steps. B takes something and eats it** | **65** | **A给B某个东西，B拿某个东西吃。** |
|  | COM -1 | A offers something to B. B takes something and tastes it | 10 | **A让B走过来，B拿某个东西吃。** |
|  | COM -2 | A asks B to move over. B takes something and eats it | 5 | **A前后走了几步，B拿某个东西吃。** |
|  | IND -1 | A turns around. B takes something and eats it | 0 | **A转身，B拿某个东西吃。** |
|  | IND-2 | A pushes something. B takes something and eats it | 20 | **A推某物，B拿某个东西吃。** |
| Look under the foot | **Com vs Ind %** |  | **80** |  |
|  | **Correct (IND)** | **A looks under his foot. B moves something** | **85** | **A让B移动某物，B移动某物。** |
|  | COM -1 | A asks B to move something. B moves something | 5 | **A让B看某物，B移动某物。** |
|  | COM -2 | A asks B to look at something. B moves something | 5 | **A看自己的脚，B移动某物。** |
|  | IND -1 | A kicks something. B moves something | 5 | **A踢某物，B移动某物。** |
|  | IND-2 | A turns around. B moves something | 0 | **A转身，B移动某物。** |
| Sneeze | **Com vs Ind %** |  | **55** |  |
|  | **Correct (IND)** | **A sneezes. B turns around** | **70** | **A让B看某物，B转身。** |
|  | COM -1 | A asks B to look at something. B turns around | 5 | **A让B坐下，B转身。** |
|  | COM -2 | A asks B to sit down. B turns around | 5 | **A打喷嚏，B转身。** |
|  | IND -1 | A eats something. B turns around | 15 | **A吃东西，B转身。** |
|  | IND-2 | A throws something on the ground. B turns around | 5 | **A向地上扔东西，B转身。** |
| Stretch | **Com vs Ind %** |  | **70** |  |
|  | **Correct (IND)** | **A stretches. B moves something** | **40** | **A让B移动某物，B移动某物。** |
|  | COM -1 | A asks B to move something. B moves something | 5 | **A让B蹲下，B移动某物。** |
|  | COM -2 | A asks B to squat down. B moves something | 0 | **A伸展，B移动某物。** |
|  | IND -1 | A picks something up. B moves something | 45 | **A捡起某物，B移动某物。** |
|  | IND-2 | A moves something. B moves something | 10 | **A移动某物，B移动某物。** |
| Turn over | **Com vs Ind %** |  | **80** |  |
|  | **Correct (IND)** | **A turns over. B squats down** | **95** | **A给B看某物，B蹲下。** |
|  | COM -1 | A shows something to B. squats down | 0 | **A让B走开，B蹲下。** |
|  | COM -2 | A asks B to move away. B squats down | 0 | **A转身，B蹲下。** |
|  | IND -1 | A moves something. B squats down | 0 | **A移动某物，B蹲下。** |
|  | IND-2 | A moves away. B squats down | 5 | **A走开，B蹲下。** |
