# Supplementary material for: The Multilingual CID-5: A New Tool to Study the Perception of Communicative Interactions in Different Languages
Source: Front Psychol. 2015 Nov 17;6:1724. doi: 10.3389/fpsyg.2015.01724 (PMC4648072; doi:10.3389/fpsyg.2015.01724)
Supplement: Supplementary file 2 [file Data_Sheet_1.ZIP › Alternatives/dutch.doc]

| **Action** | **Alternative** | **Alternatives** | **Dutch**  **% (N=20)** |  |
| --- | --- | --- | --- | --- |
| Choose which one | **Com vs Ind %** |  | **100** |  |
|  | **Correct (COM)** | **A asks B to choose between two objects. B takes an object** | **60** | **A vraagt B te kiezen tussen objecten. B kiest een object.** |
|  | COM -1 | A offers something to B. B takes an object | 20 | A biedt iets aan B aan. B kiest een object. |
|  | COM -2 | A squats down and asks B to imitate him. B takes an object | 0 | A hurkt en vraagt B hem te imiteren. B kiest een object. |
|  | IND -1 | A lifts something. B takes an object | 15 | A tilt iets op. B kiest een object. |
|  | IND-2 | A weights something in his hands. B takes an object | 5 | A weegt iets in zijn handen. B kiest een object. |
| Come closer | **Com vs Ind %** |  | **100** |  |
|  | **Correct (COM)** | **A asks B to come closer. B moves forward** | **75** | **A vraagt B om dichterbij te komen. B komt naar voren.** |
|  | COM -1 | A shows something to B. B moves forward | 10 | A toont iets aan B. B komt naar voren. |
|  | COM -2 | A waves to B. B moves forward | 15 | A wuift naar B. B komt naar voren. |
|  | IND -1 | A drinks. B moves forward | 0 | A drinkt. B komt naar voren. |
|  | IND-2 | A stretches. B moves forward | 0 | A rekt zich uit. B komt naar voren. |
| Go out of the way | **Com vs Ind %** |  | **100** |  |
|  | **Correct (COM)** | **A asks B to go out of the way. B moves over** | **30** | **A vraagt B uit de weg te gaan. B komt dichterbij.** |
|  | COM -1 | A say hello to B. B moves over | 45 | A zegt hallo tegen B. B komt dichterbij. |
|  | COM -2 | A asks B to hand him something. B moves over | 25 | A vraagt B hem iets te geven. B komt dichterbij. |
|  | IND -1 | A scratches himself. B moves over | 0 | A krabt zichzelf. B komt dichterbij. |
|  | IND-2 | A cleans something. B moves over | 0 | A ruimt iets op. B komt dichterbij. |
| Imitate me | **Com vs Ind %** |  | **100** |  |
|  | **Correct (COM)** | **A squats down, and asks B to imitate him. B squats down** | **95** | **A hurkt en vaagt B hem te imiteren. B hurkt.** |
|  | COM -1 | A gives something to B. B squats down | 0 | A geeft iets aan B. B hurkt. |
|  | COM -2 | A asks B to come closer. B squats down | 5 | A vraagt B om dichterbij te komen. B hurkt. |
|  | IND -1 | A sits down. B squats down | 0 | A gaat zitten. B hurkt. |
|  | IND-2 | A puts something down. B squats down | 0 | A zet iets neer. B hurkt. |
| Look at the ceiling | **Com vs Ind %** |  | **75** |  |
|  | **Correct (COM)** | **A asks B to look at something behind him on the ceiling. B turns around** | **50** | **A vraagt B te kijken naar iets achter hem op het plafond. B draait zich om.** |
|  | COM -1 | A asks B to turn. B turns around | 15 | A vraagt B zich om te draaien. B draait zich om. |
|  | COM -2 | A asks B to come closer. B turns around | 15 | A vraagt B om dichterbij te komen. B draait zich om. |
|  | IND -1 | A drinks. B turns around | 5 | A drinkt. B draait zich om. |
|  | IND-2 | A puts something down. B turns around | 15 | A zet iets neer. B draait zich om. |
| Look at the ground | **Com vs Ind %** |  | **95** |  |
|  | **Correct (COM)** | **A asks B to look at something on the ground. B squats down** | **70** | **A vraagt B naar iets op de grond te kijken. B hurkt.** |
|  | COM -1 | A squats down and asks B to imitate him. B squats down | 25 | A hurkt en vraagt B hem te imiteren. B hurkt. |
|  | COM -2 | A asks B to move away. B squats down | 0 | A vraagt B weg te gaan. B hurkt. |
|  | IND -1 | A sits down. B squats down | 5 | A gaat zitten. B hurkt. |
|  | IND-2 | A picks something up. B squats down | 0 | A raapt iets op. B hurkt. |
| Move this down | **Com vs Ind %** |  | **80** |  |
|  | **Correct (COM)** | **A asks B to move something. B moves something** | **75** | **A vraagt B iets te verplaatsen. B verplaatst iets.** |
|  | COM -1 | A asks B to imitate him. B moves something | 0 | A vraagt B hem te imiteren. B verplaatst iets. |
|  | COM -2 | A asks B to squat down. B moves something | 0 | A vraagt B te hurken. B verplaatst iets. |
|  | IND -1 | A pours something. B moves something | 20 | A schenkt iets uit. B verplaatst iets. |
|  | IND-2 | A moves something. B moves something | 5 | A verplaatst iets. B verplaatst iets. |
| No | **Com vs Ind %** |  | **95** |  |
|  | **Correct (COM)** | **A says ‘No’. B stops** | **85** | **A zegt 'nee'. B stopt.** |
|  | COM -1 | A says ‘Hi’. B stops | 5 | A zegt 'hallo'. B stopt. |
|  | COM -2 | A asks B to get out of the way. B stops | 10 | A vraagt B uit de weg te gaan. B stopt. |
|  | IND -1 | A rubs something. B stops | 0 | A wrijft iets. B stopt. |
|  | IND-2 | A shakes something. B stops | 0 | A schudt iets. B stopt. |
| Pick this up | **Com vs Ind %** |  | **85** |  |
|  | **Correct (COM)** | **A points to B something to pick up. B picks something up** | **90** | **A wijst iets aan om op te rapen. B raapt iets op.** |
|  | COM -1 | A asks B to squat down. B picks something up | 0 | A vraagt bij te hurken. B raapt iets op. |
|  | COM -2 | A asks B to stop. B picks something up | 0 | A vraagt B te stoppen. B raapt iets op. |
|  | IND -1 | A moves something. B picks something up | 0 | A verplaatst iets. B raapt iets op. |
|  | IND-2 | A checks the time. B picks something up | 10 | A controleert de tijd. B raapt iets op. |
| Sit down | **Com vs Ind %** |  | **85** |  |
|  | **Correct (COM)** | **A asks B to sit down. B sits down** | **80** | **A vraagt B te gaan zitten. B gaat zitten.** |
|  | COM -1 | A asks B to move over. B sits down | 10 | A vraagt B dichterbij te komen. B gaat zitten. |
|  | COM -2 | A gives something to B. B sits down | 0 | A geeft iets aan B. B gaat zitten. |
|  | IND -1 | A puts something down. B sits down | 5 | A zet iets neer. B gaat zitten. |
|  | IND-2 | A folds something. B sits down | 5 | A vouwt iets op. B gaat zitten. |
| Squat down | **Com vs Ind %** |  | **85** |  |
|  | **Correct (COM)** | **A asks B to squat down. B squats down** | **80** | **A vraagt B te hurken. B hurkt.** |
|  | COM -1 | A asks B to pick something up. B squats down | 5 | A vraagt B iets op te rapen. B hurkt. |
|  | COM -2 | A asks B to walk away. B squats down | 0 | A vraagt B weg te gaan. B hurkt. |
|  | IND -1 | A cleans something. B squats down | 5 | A ruimt iets op. B hurkt. |
|  | IND-2 | A bounces a ball. B squats down | 10 | A stuitert een bal. B hurkt. |
| Stand up | **Com vs Ind %** |  | **95** |  |
|  | **Correct (COM)** | **A asks B to stand up. B stands up** | **95** | **A vraagt B om recht te staan. B staat op.** |
|  | COM -1 | A shows something to B. B stands up | 5 | A toont iets aan B. B staat op. |
|  | COM -2 | A asks B to squat down. B stands up | 0 | A vraagt B te hurken. B staat op. |
|  | IND -1 | A bounces a ball. B stands up | 0 | A stuitert een bal. B staat op. |
|  | IND-2 | A paints something. B stands up | 0 | A schildert iets. B staat op. |
| Stop | **Com vs Ind %** |  | **85** |  |
|  | **Correct (COM)** | **A asks B to stop. B stops** | **90** | **A vraagt B te stoppen. B stopt.** |
|  | COM -1 | A shows something to B. B stops | 5 | A toont iets aan B. B stopt. |
|  | COM -2 | A asks B to sit down. B stops | 5 | A vraagt B te gaan zitten. B stopt. |
|  | IND -1 | A drinks. B stops | 0 | A drinkt. B stopt. |
|  | IND-2 | A puts something down. B stops | 0 | A zet iets neer. B stopt. |
| Walk away | **Com vs Ind %** |  | **80** |  |
|  | **Correct (COM)** | **A asks B to walk away. B takes some steps** | **40** | **A vraagt B weg te gaan. B neemt enkele stappen.** |
|  | COM -1 | A opens the door for B. B takes some steps | 35 | A opent een deur voor B. B neemt enkele stappen. |
|  | COM -2 | A asks B to move something. B takes some steps | 15 | A vraagt B iets te verplaatsen. B neemt enkele stappen. |
|  | IND -1 | A stretches. B takes some steps | 5 | A rekt zich uit. B neemt enkele stappen. |
|  | IND-2 | A draws a line. B takes some steps | 5 | A tekent een lijn. B neemt enkele stappen. |
| Drink | **Com vs Ind %** |  | **95** |  |
|  | **Correct (IND)** | **A drinks. B sits down** | **35** | **A drinkt. B gaat zitten.** |
|  | COM -1 | A asks B to sit down. B sits down | 5 | A vraagt B te gaan zitten. B gaat zitten. |
|  | COM -2 | A asks B to look at something. B sits down | 15 | A vraagt B naar iets te kijken. B gaat zitten. |
|  | IND -1 | A looks at the time. B sits down | 15 | A controleert de tijd. B gaat zitten. |
|  | IND-2 | A scratches his head. B sits down | 30 | A krabt zijn hoofd. B gaat zitten. |
| Jump | **Com vs Ind %** |  | **100** |  |
|  | **Correct (IND)** | **A jumps. B picks something up** | **100** | **A springt. B raapt iets op.** |
|  | COM -1 | A tells B he is very happy. B picks something up | 0 | A vertelt aan B hoe blij hij is. B raapt iets op. |
|  | COM -2 | A asks B to pick something up. B picks something up | 0 | A vraagt B iets op te rapen. B raapt iets op. |
|  | IND -1 | A moves over. B picks something up | 0 | A komt dichterbij. B raapt iets op. |
|  | IND-2 | A lifts something. B picks something up | 0 | A tilt iets op. B raapt iets op. |
| Lateral steps | **Com vs Ind %** |  | **90** |  |
|  | **Correct (IND)** | **A makes some lateral steps. B takes something and eats it** | **80** | **A neemte enkele zijwaartse stappen. B neemt iets en eet het op**. |
|  | COM -1 | A offers something to B. B takes something and tastes it | 5 | A biedt iets aan B aan. B neemt iets en eet het op. |
|  | COM -2 | A asks B to move over. B takes something and eats it | 5 | A vraag B dichterbij te komen. B neemt iets en eet het op. |
|  | IND -1 | A turns around. B takes something and eats it | 5 | A draait zich om. B neemt iets en eet het op. |
|  | IND-2 | A pushes something. B takes something and eats it | 5 | A duwt iets. B neemt iets en eet het op. |
| Look under the foot | **Com vs Ind %** |  | **95** |  |
|  | **Correct (IND)** | **A looks under his foot. B moves something** | **100** | **A kijkt onder zijn voet. B verplaats iets.** |
|  | COM -1 | A asks B to move something. B moves something | 0 | A vraagt B iets te verplaatsen. B verplaatst iets. |
|  | COM -2 | A asks B to look at something. B moves something | 0 | A vraagt B naar iets te kijken. B verplaatst iets. |
|  | IND -1 | A kicks something. B moves something | 0 | A schopt tegen iets. B verplaatst iets. |
|  | IND-2 | A turns around. B moves something | 0 | A draait zich om. B verplaatst iets. |
| Sneeze | **Com vs Ind %** |  | **75** |  |
|  | **Correct (IND)** | **A sneezes. B turns around** | **55** | **A niest. B draait zich om.** |
|  | COM -1 | A asks B to look at something. B turns around | 5 | A vraagt B naar iets te kijken. B draait zich om. |
|  | COM -2 | A asks B to sit down. B turns around | 15 | A vraagt B te gaan zitten. B draait zich om. |
|  | IND -1 | A eats something. B turns around | 5 | A eet iets. B draait zich om. |
|  | IND-2 | A throws something on the ground. B turns around | 20 | A gooit iets op de grond. B draait zich om. |
| Stretch | **Com vs Ind %** |  | **70** |  |
|  | **Correct (IND)** | **A stretches. B moves something** | **30** | **A rekt zich uit. B verplaatst iets.** |
|  | COM -1 | A asks B to move something. B moves something | 25 | A vraagt B iets te verplaatsen. B verplaatst iets. |
|  | COM -2 | A asks B to squat down. B moves something | 5 | A vraagt B te hurken. B verplaatst iets. |
|  | IND -1 | A picks something up. B moves something | 35 | A raapt iets op. B verplaatst iets. |
|  | IND-2 | A moves something. B moves something | 5 | A verplaatst iets. B verplaatst iets. |
| Turn over | **Com vs Ind %** |  | **100** |  |
|  | **Correct (IND)** | **A turns over. B squats down** | **90** | **A draait zich om. B bukt zich.** |
|  | COM -1 | A shows something to B. squats down | 5 | A toont iets aan B. B bukt zich. |
|  | COM -2 | A asks B to move away. B squats down | 0 | A vraagt B weg te gaan. B bukt zich. |
|  | IND -1 | A moves something. B squats down | 0 | A verplaatst iets. B bukt zich. |
|  | IND-2 | A moves away. B squats down | 5 | A gaat weg. B bukt zich. |
