# Supplementary material for: The Multilingual CID-5: A New Tool to Study the Perception of Communicative Interactions in Different Languages
Source: Front Psychol. 2015 Nov 17;6:1724. doi: 10.3389/fpsyg.2015.01724 (PMC4648072; doi:10.3389/fpsyg.2015.01724)
Supplement: Supplementary file 2 [file Data_Sheet_1.ZIP › Alternatives/english.doc]

| **Action** | **Alternative** | **Alternatives** | **English**  **% (N=20)** |
| --- | --- | --- | --- |
| Choose which one | **Com vs Ind %** |  | **100** |
|  | **Correct (COM)** | **A asks B to choose between two objects. B takes an object** | **50** |
|  | COM -1 | A offers something to B. B takes an object | 35 |
|  | COM -2 | A squats down and asks B to imitate him. B takes an object | 0 |
|  | IND -1 | A lifts something. B takes an object | 5 |
|  | IND-2 | A weights something in his hands. B takes an object | 10 |
| Come closer | **Com vs Ind %** |  | **100** |
|  | **Correct (COM)** | **A asks B to come closer. B moves forward** | **85** |
|  | COM -1 | A shows something to B. B moves forward | 0 |
|  | COM -2 | A waves to B. B moves forward | 15 |
|  | IND -1 | A drinks. B moves forward | 0 |
|  | IND-2 | A stretches. B moves forward | 0 |
| Go out of the way | **Com vs Ind %** |  | **100** |
|  | **Correct (COM)** | **A asks B to go out of the way. B moves over** | **60** |
|  | COM -1 | A say hello to B. B moves over | 20 |
|  | COM -2 | A asks B to hand him something. B moves over | 20 |
|  | IND -1 | A scratches himself. B moves over | 0 |
|  | IND-2 | A cleans something. B moves over | 0 |
| Imitate me | **Com vs Ind %** |  | **95** |
|  | **Correct (COM)** | **A squats down, and asks B to imitate him. B squats down** | **95** |
|  | COM -1 | A gives something to B. B squats down | 0 |
|  | COM -2 | A asks B to come closer. B squats down | 0 |
|  | IND -1 | A sits down. B squats down | 0 |
|  | IND-2 | A puts something down. B squats down | 5 |
| Look at the ceiling | **Com vs Ind %** |  | **70** |
|  | **Correct (COM)** | **A asks B to look at something behind him on the ceiling. B turns around** | **55** |
|  | COM -1 | A asks B to turn. B turns around | 20 |
|  | COM -2 | A asks B to come closer. B turns around | 0 |
|  | IND -1 | A drinks. B turns around | 25 |
|  | IND-2 | A puts something down. B turns around | 0 |
| Look at the ground | **Com vs Ind %** |  | **95** |
|  | **Correct (COM)** | **A asks B to look at something on the ground. B squats down** | **70** |
|  | COM -1 | A squats down and asks B to imitate him. B squats down | 10 |
|  | COM -2 | A asks B to move away. B squats down | 0 |
|  | IND -1 | A sits down. B squats down | 20 |
|  | IND-2 | A picks something up. B squats down | 0 |
| Move this down | **Com vs Ind %** |  | **90** |
|  | **Correct (COM)** | **A asks B to move something. B moves something** | **90** |
|  | COM -1 | A asks B to imitate him. B moves something | 0 |
|  | COM -2 | A asks B to squat down. B moves something | 0 |
|  | IND -1 | A pours something. B moves something | 5 |
|  | IND-2 | A moves something. B moves something | 5 |
| No | **Com vs Ind %** |  | **95** |
|  | **Correct (COM)** | **A says ‘No’. B stops** | **90** |
|  | COM -1 | A says ‘Hi’. B stops | 10 |
|  | COM -2 | A asks B to get out of the way. B stops | 0 |
|  | IND -1 | A rubs something. B stops | 0 |
|  | IND-2 | A shakes something. B stops | 0 |
| Pick this up | **Com vs Ind %** |  | **95** |
|  | **Correct (COM)** | **A points to B something to pick up. B picks something up** | **95** |
|  | COM -1 | A asks B to squat down. B picks something up | 0 |
|  | COM -2 | A asks B to stop. B picks something up | 0 |
|  | IND -1 | A moves something. B picks something up | 5 |
|  | IND-2 | A checks the time. B picks something up | 0 |
| Sit down | **Com vs Ind %** |  | **90** |
|  | **Correct (COM)** | **A asks B to sit down. B sits down** | **90** |
|  | COM -1 | A asks B to move over. B sits down | 0 |
|  | COM -2 | A gives something to B. B sits down | 0 |
|  | IND -1 | A puts something down. B sits down | 0 |
|  | IND-2 | A folds something. B sits down | 10 |
| Squat down | **Com vs Ind %** |  | **95** |
|  | **Correct (COM)** | **A asks B to squat down. B squats down** | **75** |
|  | COM -1 | A asks B to pick something up. B squats down | 10 |
|  | COM -2 | A asks B to walk away. B squats down | 0 |
|  | IND -1 | A cleans something. B squats down | 0 |
|  | IND-2 | A bounces a ball. B squats down | 15 |
| Stand up | **Com vs Ind %** |  | **95** |
|  | **Correct (COM)** | **A asks B to stand up. B stands up** | **80** |
|  | COM -1 | A shows something to B. B stands up | 5 |
|  | COM -2 | A asks B to squat down. B stands up | 0 |
|  | IND -1 | A bounces a ball. B stands up | 5 |
|  | IND-2 | A paints something. B stands up | 10 |
| Stop | **Com vs Ind %** |  | **95** |
|  | **Correct (COM)** | **A asks B to stop. B stops** | **90** |
|  | COM -1 | A shows something to B. B stops | 5 |
|  | COM -2 | A asks B to sit down. B stops | 0 |
|  | IND -1 | A drinks. B stops | 5 |
|  | IND-2 | A puts something down. B stops | 0 |
| Walk away | **Com vs Ind %** |  | **90** |
|  | **Correct (COM)** | **A asks B to walk away. B takes some steps** | **30** |
|  | COM -1 | A opens the door for B. B takes some steps | 55 |
|  | COM -2 | A asks B to move something. B takes some steps | 5 |
|  | IND -1 | A stretches. B takes some steps | 10 |
|  | IND-2 | A draws a line. B takes some steps | 0 |
| Drink | **Com vs Ind %** |  | **90** |
|  | **Correct (IND)** | **A drinks. B sits down** | **85** |
|  | COM -1 | A asks B to sit down. B sits down | 5 |
|  | COM -2 | A asks B to look at something. B sits down | 5 |
|  | IND -1 | A looks at the time. B sits down | 0 |
|  | IND-2 | A scratches his head. B sits down | 5 |
| Jump | **Com vs Ind %** |  | **100** |
|  | **Correct (IND)** | **A jumps. B picks something up** | **100** |
|  | COM -1 | A tells B he is very happy. B picks something up | 0 |
|  | COM -2 | A asks B to pick something up. B picks something up | 0 |
|  | IND -1 | A moves over. B picks something up | 0 |
|  | IND-2 | A lifts something. B picks something up | 0 |
| Lateral steps | **Com vs Ind %** |  | **85** |
|  | **Correct (IND)** | **A makes some lateral steps. B takes something and eats it** | **85** |
|  | COM -1 | A offers something to B. B takes something and tastes it | 15 |
|  | COM -2 | A asks B to move over. B takes something and eats it | 0 |
|  | IND -1 | A turns around. B takes something and eats it | 0 |
|  | IND-2 | A pushes something. B takes something and eats it | 0 |
| Look under the foot | **Com vs Ind %** |  | **100** |
|  | **Correct (IND)** | **A looks under his foot. B moves something** | **95** |
|  | COM -1 | A asks B to move something. B moves something | 0 |
|  | COM -2 | A asks B to look at something. B moves something | 0 |
|  | IND -1 | A kicks something. B moves something | 5 |
|  | IND-2 | A turns around. B moves something | 0 |
| Sneeze | **Com vs Ind %** |  | **70** |
|  | **Correct (IND)** | **A sneezes. B turns around** | **85** |
|  | COM -1 | A asks B to look at something. B turns around | 0 |
|  | COM -2 | A asks B to sit down. B turns around | 0 |
|  | IND -1 | A eats something. B turns around | 5 |
|  | IND-2 | A throws something on the ground. B turns around | 10 |
| Stretch | **Com vs Ind %** |  | **90** |
|  | **Correct (IND)** | **A stretches. B moves something** | **60** |
|  | COM -1 | A asks B to move something. B moves something | 15 |
|  | COM -2 | A asks B to squat down. B moves something | 0 |
|  | IND -1 | A picks something up. B moves something | 25 |
|  | IND-2 | A moves something. B moves something | 0 |
| Turn over | **Com vs Ind %** |  | **100** |
|  | **Correct (IND)** | **A turns over. B squats down** | **75** |
|  | COM -1 | A shows something to B. squats down | 0 |
|  | COM -2 | A asks B to move away. B squats down | 0 |
|  | IND -1 | A moves something. B squats down | 0 |
|  | IND-2 | A moves away. B squats down | 25 |
