# Supplementary material for: The Multilingual CID-5: A New Tool to Study the Perception of Communicative Interactions in Different Languages
Source: Front Psychol. 2015 Nov 17;6:1724. doi: 10.3389/fpsyg.2015.01724 (PMC4648072; doi:10.3389/fpsyg.2015.01724)
Supplement: Supplementary file 2 [file Data_Sheet_1.ZIP › Alternatives/french.doc]

| **Action** | **Alternative** | **Alternatives** | **French**  **% (N=20)** |  |
| --- | --- | --- | --- | --- |
| Choose which one | **Com vs Ind %** |  | **100** |  |
|  | **Correct (COM)** | **A asks B to choose between two objects. B takes an object** | **55** | **A demande à B de choisir entre deux objets. B prend un objet** |
|  | COM -1 | A offers something to B. B takes an object | 35 | A offre quelque chose à B. B prend un objet |
|  | COM -2 | A squats down and asks B to imitate him. B takes an object | 0 | A s’accroupit et demande à B de l’imiter. B prend un objet |
|  | IND -1 | A lifts something. B takes an object | 5 | A soulève quelque chose. B prend un objet |
|  | IND-2 | A weights something in his hands. B takes an object | 5 | A soupèse quelque chose dans ses mains. B prend un objet |
| Come closer | **Com vs Ind %** |  | **95** |  |
|  | **Correct (COM)** | **A asks B to come closer. B moves forward** | **95** | **A demande à B de s’approcher. B avance** |
|  | COM -1 | A shows something to B. B moves forward | 0 | A montre quelque chose à B. B avance |
|  | COM -2 | A waves to B. B moves forward | 0 | A salue B. B avance |
|  | IND -1 | A drinks. B moves forward | 5 | A boit. B avance |
|  | IND-2 | A stretches. B moves forward | 0 | A s’étire. B avance |
| Go out of the way | **Com vs Ind %** |  | **100** |  |
|  | **Correct (COM)** | **A asks B to go out of the way. B moves over** | **90** | **A demande à B de libérer le passage. B se pousse** |
|  | COM -1 | A say hello to B. B moves over | 5 | A dit bonjour à B. B se pousse |
|  | COM -2 | A asks B to hand him something. B moves over | 0 | A demande à B de lui donner quelque chose. B se pousse |
|  | IND -1 | A scratches himself. B moves over | 0 | A se gratte. B se pousse |
|  | IND-2 | A cleans something. B moves over | 5 | A nettoie quelque chose. B se pousse |
| Imitate me | **Com vs Ind %** |  | **100** |  |
|  | **Correct (COM)** | **A squats down, and asks B to imitate him. B squats down** | **95** | **A s’accroupit, et demande à B de l’imiter. B s’accroupit** |
|  | COM -1 | A gives something to B. B squats down | 0 | A donne quelque chose à B. B s’accroupit |
|  | COM -2 | A asks B to come closer. B squats down | 5 | A demande à B de s’approcher. B s’accroupit |
|  | IND -1 | A sits down. B squats down | 0 | A s’assoit. B s’accroupit |
|  | IND-2 | A puts something down. B squats down | 0 | A pose quelque chose. B s’accroupit |
| Look at the ceiling | **Com vs Ind %** |  | **60** |  |
|  | **Correct (COM)** | **A asks B to look at something behind him on the ceiling. B turns around** | **35** | **A demande à B de regarder quelque chose derrière lui au plafond. B se retourne** |
|  | COM -1 | A asks B to turn. B turns around | 10 | A demande à B de se tourner. B se retourne |
|  | COM -2 | A asks B to come closer. B turns around | 25 | A demande à B de s’approcher. B se retourne |
|  | IND -1 | A drinks. B turns around | 30 | A boit. B se retourne |
|  | IND-2 | A puts something down. B turns around | 0 | A pose quelque chose. B se retourne |
| Look at the ground | **Com vs Ind %** |  | **100** |  |
|  | **Correct (COM)** | **A asks B to look at something on the ground. B squats down** | **65** | **A demande à B de regarder quelque chose au sol. B s’accroupit** |
|  | COM -1 | A squats down and asks B to imitate him. B squats down | 30 | A s’accroupit et demande à B de l’imiter. B s’accroupit |
|  | COM -2 | A asks B to move away. B squats down | 0 | A demande à B de s’éloigner. B s’accroupit |
|  | IND -1 | A sits down. B squats down | 0 | A s’assoit. B s’accroupit |
|  | IND-2 | A picks something up. B squats down | 5 | A ramasse quelque chose. B s’accroupit |
| Move this down | **Com vs Ind %** |  | **55** |  |
|  | **Correct (COM)** | **A asks B to move something. B moves something** | **55** | **A demande à B de déplacer quelque chose. B déplace quelque chose** |
|  | COM -1 | A asks B to imitate him. B moves something | 0 | A demande à B de l’imiter. B déplace quelque chose |
|  | COM -2 | A asks B to squat down. B moves something | 0 | A demande à B de s’accroupir. B déplace quelque chose |
|  | IND -1 | A pours something. B moves something | 40 | A verse quelque chose. B déplace quelque chose |
|  | IND-2 | A moves something. B moves something | 5 | A déplace quelque chose. B déplace quelque chose |
| No | **Com vs Ind %** |  | **100** |  |
|  | **Correct (COM)** | **A says ‘No’. B stops** | **100** | **A dit « Non ». B s’arrête** |
|  | COM -1 | A says ‘Hi’. B stops | 0 | A dit « Salut ». B s’arrête |
|  | COM -2 | A asks B to get out of the way. B stops | 0 | A demande à B de libérer le passage. B s’arrête |
|  | IND -1 | A rubs something. B stops | 0 | A frotte quelque chose. B s’arrête |
|  | IND-2 | A shakes something. B stops | 0 | A secoue quelque chose. B s’arrête |
| Pick this up | **Com vs Ind %** |  | **80** |  |
|  | **Correct (COM)** | **A points to B something to pick up. B picks something up** | **85** | **A montre à B quelque chose à ramasser. B ramasse quelque chose** |
|  | COM -1 | A asks B to squat down. B picks something up | 0 | A demande à B de s’accroupir. B ramasse quelque chose |
|  | COM -2 | A asks B to stop. B picks something up | 0 | A demande à B de s’arrêter. B ramasse quelque chose |
|  | IND -1 | A moves something. B picks something up | 15 | A déplace quelque chose. B ramasse quelque chose |
|  | IND-2 | A checks the time. B picks something up | 0 | A regarde l’heure. B ramasse quelque chose |
| Sit down | **Com vs Ind %** |  | **95** |  |
|  | **Correct (COM)** | **A asks B to sit down. B sits down** | **95** | **A demande à B de s’asseoir. B s’assied** |
|  | COM -1 | A asks B to move over. B sits down | 0 | A demande à B de se pousser. B s’assied |
|  | COM -2 | A gives something to B. B sits down | 0 | A donne quelque chose à B. B s’assied |
|  | IND -1 | A puts something down. B sits down | 5 | A pose quelque chose. B s’assied |
|  | IND-2 | A folds something. B sits down | 0 | A plie quelque chose. B s’assied |
| Squat down | **Com vs Ind %** |  | **95** |  |
|  | **Correct (COM)** | **A asks B to squat down. B squats down** | **95** | **A demande à B de s’accroupir. B s’accroupit** |
|  | COM -1 | A asks B to pick something up. B squats down | 0 | A demande à B de ramasser quelque chose. B s’accroupit |
|  | COM -2 | A asks B to walk away. B squats down | 0 | A demande à B de s’éloigner. B s’accroupit |
|  | IND -1 | A cleans something. B squats down | 5 | A nettoie quelque chose. B s’accroupit |
|  | IND-2 | A bounces a ball. B squats down | 0 | A fait rebondir une balle. B s’accroupit |
| Stand up | **Com vs Ind %** |  | **100** |  |
|  | **Correct (COM)** | **A asks B to stand up. B stands up** | **95** | **A demande à B de se lever. B se lève** |
|  | COM -1 | A shows something to B. B stands up | 0 | A montre quelque chose à B. B se lève |
|  | COM -2 | A asks B to squat down. B stands up | 0 | A demande à B de s’accroupir. B se lève |
|  | IND -1 | A bounces a ball. B stands up | 0 | A fait rebondir une balle. B se lève |
|  | IND-2 | A paints something. B stands up | 5 | A peint quelque chose. B se lève |
| Stop | **Com vs Ind %** |  | **95** |  |
|  | **Correct (COM)** | **A asks B to stop. B stops** | **100** | **A demande à B de s’arrêter. B s’arrête** |
|  | COM -1 | A shows something to B. B stops | 0 | A montre quelque chose à B. B s’arrête |
|  | COM -2 | A asks B to sit down. B stops | 0 | A demande à B de s’asseoir. B s’arrête |
|  | IND -1 | A drinks. B stops | 0 | A boit. B s’arrête |
|  | IND-2 | A puts something down. B stops | 0 | A pose quelque chose. B s’arrête |
| Walk away | **Com vs Ind %** |  | **60** |  |
|  | **Correct (COM)** | **A asks B to walk away. B takes some steps** | **25** | **A demande à B de s’éloigner. B fait quelques pas** |
|  | COM -1 | A opens the door for B. B takes some steps | 55 | A ouvre la porte pour B. B fait quelques pas |
|  | COM -2 | A asks B to move something. B takes some steps | 5 | A demande à B de déplacer quelque chose. B fait quelques pas |
|  | IND -1 | A stretches. B takes some steps | 15 | A s’étire. B fait quelques pas |
|  | IND-2 | A draws a line. B takes some steps | 0 | A trace une ligne. B fait quelques pas |
| Drink | **Com vs Ind %** |  | **100** |  |
|  | **Correct (IND)** | **A drinks. B sits down** | **95** | **A boit. B s’assied** |
|  | COM -1 | A asks B to sit down. B sits down | 0 | A demande à B de s’asseoir. B s’assied |
|  | COM -2 | A asks B to look at something. B sits down | 0 | A demande à B de regarder quelque chose. B s’assied |
|  | IND -1 | A looks at the time. B sits down | 0 | A regarde l’heure. B s’assied |
|  | IND-2 | A scratches his head. B sits down | 5 | A se gratte la tête. B s’assied |
| Jump | **Com vs Ind %** |  | **100** |  |
|  | **Correct (IND)** | **A jumps. B picks something up** | **95** | **A saute. B ramasse quelque chose** |
|  | COM -1 | A tells B he is very happy. B picks something up | 0 | A dit à B qu’il est très heureux. B ramasse quelque chose |
|  | COM -2 | A asks B to pick something up. B picks something up | 0 | A demande à B de ramasser quelque chose. B ramasse quelque chose |
|  | IND -1 | A moves over. B picks something up | 0 | A se pousse. B ramasse quelque chose |
|  | IND-2 | A lifts something. B picks something up | 5 | A soulève quelque chose. B ramasse quelque chose |
| Lateral steps | **Com vs Ind %** |  | **90** |  |
|  | **Correct (IND)** | **A makes some lateral steps. B takes something and eats it** | **80** | **A fait quelques pas sur le côté. B prend quelque chose et le mange** |
|  | COM -1 | A offers something to B. B takes something and tastes it | 0 | A offre quelque chose à B. B prend quelque chose et le mange |
|  | COM -2 | A asks B to move over. B takes something and eats it | 5 | A demande à B de se pousser. B prend quelque chose et le mange |
|  | IND -1 | A turns around. B takes something and eats it | 0 | A se retourne. B prend quelque chose et le mange |
|  | IND-2 | A pushes something. B takes something and eats it | 15 | A pousse quelque chose. B prend quelque chose et le mange |
| Look under the foot | **Com vs Ind %** |  | **95** |  |
|  | **Correct (IND)** | **A looks under his foot. B moves something** | **95** | **A regarde sous son pied. B déplace quelque chose** |
|  | COM -1 | A asks B to move something. B moves something | 0 | A demande à B de déplacer quelque chose. B déplace quelque chose |
|  | COM -2 | A asks B to look at something. B moves something | 0 | A demande à B de regarder quelque chose. B déplace quelque chose |
|  | IND -1 | A kicks something. B moves something | 0 | A donne un coup de pied dans quelque chose. B déplace quelque chose |
|  | IND-2 | A turns around. B moves something | 5 | A se retourne. B déplace quelque chose |
| Sneeze | **Com vs Ind %** |  | **75** |  |
|  | **Correct (IND)** | **A sneezes. B turns around** | **85** | **A éternue. B se retourne** |
|  | COM -1 | A asks B to look at something. B turns around | 0 | A demande à B de regarder quelque chose. B se retourne |
|  | COM -2 | A asks B to sit down. B turns around | 5 | A demande à B de s’asseoir. B se retourne |
|  | IND -1 | A eats something. B turns around | 0 | A mange quelque chose. B se retourne |
|  | IND-2 | A throws something on the ground. B turns around | 10 | A jette quelque chose au sol. B se retourne |
| Stretch | **Com vs Ind %** |  | **95** |  |
|  | **Correct (IND)** | **A stretches. B moves something** | **65** | **A fait des étirements. B déplace quelque chose** |
|  | COM -1 | A asks B to move something. B moves something | 10 | A demande à B de déplacer quelque chose. B déplace quelque chose |
|  | COM -2 | A asks B to squat down. B moves something | 0 | A demande à B de s’accroupir. B déplace quelque chose |
|  | IND -1 | A picks something up. B moves something | 25 | A ramasse quelque chose. B déplace quelque chose |
|  | IND-2 | A moves something. B moves something | 0 | A déplace quelque chose. B déplace quelque chose |
| Turn over | **Com vs Ind %** |  | **100** |  |
|  | **Correct (IND)** | **A turns over. B squats down** | **100** | **A se retourne. B se penche** |
|  | COM -1 | A shows something to B. squats down | 0 | A montre quelque chose à B. B se penche |
|  | COM -2 | A asks B to move away. B squats down | 0 | A demande à B de s’éloigner. B se penche |
|  | IND -1 | A moves something. B squats down | 0 | A déplace quelque chose. B se penche |
|  | IND-2 | A moves away. B squats down | 0 | A s’éloigne. B se penche |
