# Supplementary material for: The Multilingual CID-5: A New Tool to Study the Perception of Communicative Interactions in Different Languages
Source: Front Psychol. 2015 Nov 17;6:1724. doi: 10.3389/fpsyg.2015.01724 (PMC4648072; doi:10.3389/fpsyg.2015.01724)
Supplement: Supplementary file 2 [file Data_Sheet_1.ZIP › Alternatives/german.doc]

| **Action** | **Alternative** | **Alternatives** | **German**  **% (N=20)** |  |
| --- | --- | --- | --- | --- |
| Choose which one | **Com vs Ind %** |  | **100** |  |
|  | **Correct (COM)** | **A asks B to choose between two objects. B takes an object** | **80** | **A bittet B sich einen von zwei Gegenständen auszusuchen. B nimmt einen Gegenstand** |
|  | COM -1 | A offers something to B. B takes an object | 20 | A bietet B etwas an. B nimmt einen Gegenstand |
|  | COM -2 | A squats down and asks B to imitate him. B takes an object | 0 | A geht in die Hocke und bittet B ihn nachzuahmen. B nimmt einen Gegenstand |
|  | IND -1 | A lifts something. B takes an object | 0 | A hebt etwas. B nimmt einen Gegenstand |
|  | IND-2 | A weights something in his hands. B takes an object | 0 | A wiegt etwas in seiner Hand. B nimmt einen Gegenstand |
| Come closer | **Com vs Ind %** |  | **90** |  |
|  | **Correct (COM)** | **A asks B to come closer. B moves forward** | **70** | **A bittet B näher zu kommen. B geht nach vorne** |
|  | COM -1 | A shows something to B. B moves forward | 10 | A zeigt B etwas. B geht nach vorne |
|  | COM -2 | A waves to B. B moves forward | 15 | A winkt B zu. B geht nach vorne |
|  | IND -1 | A drinks. B moves forward | 0 | A winkt B zu. B geht nach vorne |
|  | IND-2 | A stretches. B moves forward | 5 | A macht Dehnübungen. B geht nach vorne |
| Go out of the way | **Com vs Ind %** |  | **100** |  |
|  | **Correct (COM)** | **A asks B to go out of the way. B moves over** | **65** | **A bittet B aus dem Weg zu gehen. B geht zur Seite** |
|  | COM -1 | A say hello to B. B moves over | 20 | A grüßt B. B geht zur Seite |
|  | COM -2 | A asks B to hand him something. B moves over | 5 | A bittet B ihm etwas zu reichen. B geht zur Seite |
|  | IND -1 | A scratches himself. B moves over | 10 | A kratzt sich. B geht zur Seite |
|  | IND-2 | A cleans something. B moves over | 0 | A reinigt etwas. B geht zur Seite |
| Imitate me | **Com vs Ind %** |  | **100** |  |
|  | **Correct (COM)** | **A squats down, and asks B to imitate him. B squats down** | **100** | **A geht in die Hocke und bittet B ihn nachzuahmen. B geht in die Hocke** |
|  | COM -1 | A gives something to B. B squats down | 0 | A gibt B etwas. B geht in die Hocke |
|  | COM -2 | A asks B to come closer. B squats down | 0 | A bittet B näher zu kommen. B geht in die Hocke |
|  | IND -1 | A sits down. B squats down | 0 | A setzt sich. B geht in die Hocke |
|  | IND-2 | A puts something down. B squats down | 0 | A legt etwas nieder. B geht in die Hocke |
| Look at the ceiling | **Com vs Ind %** |  | **70** |  |
|  | **Correct (COM)** | **A asks B to look at something behind him on the ceiling. B turns around** | **55** | **A bittet B sich etwas hinter ihm an der Decke anzuschauen. B dreht sich um** |
|  | COM -1 | A asks B to turn. B turns around | 20 | A bittet B sich zu drehen. B dreht sich um |
|  | COM -2 | A asks B to come closer. B turns around | 15 | A bittet B naeher zu kommen. B dreht sich um |
|  | IND -1 | A drinks. B turns around | 10 | A trinkt. B dreht sich um |
|  | IND-2 | A puts something down. B turns around | 0 | A legt etwas nieder. B dreht sich um |
| Look at the ground | **Com vs Ind %** |  | **100** |  |
|  | **Correct (COM)** | **A asks B to look at something on the ground. B squats down** | **65** | **A bittet B etwas am Boden anzuschauen. B geht in die Hocke** |
|  | COM -1 | A squats down and asks B to imitate him. B squats down | 30 | A geht in die Hocke und bittet B ihn nachzuahmen. B geht in die Hocke |
|  | COM -2 | A asks B to move away. B squats down | 0 | A bittet B aus dem wegzugehen. B geht in die Hocke |
|  | IND -1 | A sits down. B squats down | 5 | A setzt sich. B geht in die Hocke |
|  | IND-2 | A picks something up. B squats down | 0 | A hebt etwas auf. B geht in die Hocke |
| Move this down | **Com vs Ind %** |  | **90** |  |
|  | **Correct (COM)** | **A asks B to move something. B moves something** | **95** | **A bittet B etwas zu bewegen. B bewegt etwas** |
|  | COM -1 | A asks B to imitate him. B moves something | 0 | A bittet B ihn nachzuahmen. B bewegt etwas |
|  | COM -2 | A asks B to squat down. B moves something | 0 | A bittet B in die Hocke zu gehen. B bewegt etwas |
|  | IND -1 | A pours something. B moves something | 5 | A gießt etwas. B bewegt etwas |
|  | IND-2 | A moves something. B moves something | 0 | A bewegt etwas. B bewegt etwas |
| No | **Com vs Ind %** |  | **95** |  |
|  | **Correct (COM)** | **A says ‘No’. B stops** | **90** | **A sagt Nein. B stoppt** |
|  | COM -1 | A says ‘Hi’. B stops | 0 | A sagt Hallo. B stoppt |
|  | COM -2 | A asks B to get out of the way. B stops | 0 | A bittet B aus dem Weg zu gehen. B stoppt |
|  | IND -1 | A rubs something. B stops | 0 | A reibt etwas. B stoppt |
|  | IND-2 | A shakes something. B stops | 10 | A schüttelt etwas. B stoppt |
| Pick this up | **Com vs Ind %** |  | **80** |  |
|  | **Correct (COM)** | **A points to B something to pick up. B picks something up** | **75** | **A zeigt B etwas zum Aufheben. B hebt etwas auf** |
|  | COM -1 | A asks B to squat down. B picks something up | 5 | A bittet B in die Hocke zu gehen. B hebt etwas auf |
|  | COM -2 | A asks B to stop. B picks something up | 0 | A bittet B zu stoppen. B hebt etwas auf |
|  | IND -1 | A moves something. B picks something up | 0 | A bewegt etwas. B hebt etwas auf |
|  | IND-2 | A checks the time. B picks something up | 20 | A schaut auf die Uhr. B hebt etwas auf |
| Sit down | **Com vs Ind %** |  | **95** |  |
|  | **Correct (COM)** | **A asks B to sit down. B sits down** | **100** | **A bittet B sich zu setzen. B setzt sich** |
|  | COM -1 | A asks B to move over. B sits down | 0 | A bittet B aus dem Weg zu gehen. B setzt sich |
|  | COM -2 | A gives something to B. B sits down | 0 | A gibt B etwas. B setzt sich |
|  | IND -1 | A puts something down. B sits down | 0 | A legt etwas nieder. B setzt sich |
|  | IND-2 | A folds something. B sits down | 0 | A faltet etwas. B setzt sich |
| Squat down | **Com vs Ind %** |  | **95** |  |
|  | **Correct (COM)** | **A asks B to squat down. B squats down** | **75** | **A bittet B in die Hocke zu gehen. B geht in die Hocke** |
|  | COM -1 | A asks B to pick something up. B squats down | 15 | A bittet B etwas aufzuheben. B geht in die Hocke down |
|  | COM -2 | A asks B to walk away. B squats down | 0 | A bittet B wegzugehen. B geht in die Hocke |
|  | IND -1 | A cleans something. B squats down | 5 | A reinigt etwas. B geht in die Hocke |
|  | IND-2 | A bounces a ball. B squats down | 5 | A lässt einen Ball springen. B geht in die Hocke |
| Stand up | **Com vs Ind %** |  | **95** |  |
|  | **Correct (COM)** | **A asks B to stand up. B stands up** | **100** | **A bittet B aufzustehen. B steht auf** |
|  | COM -1 | A shows something to B. B stands up | 0 | A zeigt B etwas. B steht auf |
|  | COM -2 | A asks B to squat down. B stands up | 0 | A bittet B in die Hocke zu gehen. B steht auf |
|  | IND -1 | A bounces a ball. B stands up | 0 | A lässt einen Ball springen. B steht auf |
|  | IND-2 | A paints something. B stands up | 0 | A malt etwas. B steht auf |
| Stop | **Com vs Ind %** |  | **100** |  |
|  | **Correct (COM)** | **A asks B to stop. B stops** | **100** | **A bittet B zu stoppen. B stoppt** |
|  | COM -1 | A shows something to B. B stops | 0 | A zeigt B etwas. B stoppt |
|  | COM -2 | A asks B to sit down. B stops | 0 | A bittet B sich zu setzen. B stoppt |
|  | IND -1 | A drinks. B stops | 0 | A trinkt. B stoppt |
|  | IND-2 | A puts something down. B stops | 0 | A legt etwas nieder. B stoppt |
| Walk away | **Com vs Ind %** |  | **70** |  |
|  | **Correct (COM)** | **A asks B to walk away. B takes some steps** | **10** | **A bittet B wegzugehen. B geht ein paar Schritte** |
|  | COM -1 | A opens the door for B. B takes some steps | 70 | A öffnet B die Tür. B geht ein paar Schritte |
|  | COM -2 | A asks B to move something. B takes some steps | 15 | A bittet B etwas zu bewegen. B geht ein paar Schritte |
|  | IND -1 | A stretches. B takes some steps | 0 | A macht Dehnübungen. B geht ein paar Schritte |
|  | IND-2 | A draws a line. B takes some steps | 5 | A zeichnet eine Linie. B geht ein paar Schritte |
| Drink | **Com vs Ind %** |  | **80** |  |
|  | **Correct (IND)** | **A drinks. B sits down** | **75** | **A trinkt. B setzt sich** |
|  | COM -1 | A asks B to sit down. B sits down | 20 | A bittet B sich zu setzen. B setzt sich |
|  | COM -2 | A asks B to look at something. B sits down | 5 | A bittet sich etwas anzuschauen. B setzt sich |
|  | IND -1 | A looks at the time. B sits down | 0 | A schaut auf die Uhr. B setzt sich |
|  | IND-2 | A scratches his head. B sits down | 0 | A kratzt sich am Kopf. B setzt sich |
| Jump | **Com vs Ind %** |  | **95** |  |
|  | **Correct (IND)** | **A jumps. B picks something up** | **95** | **A springt. B hebt etwas auf** |
|  | COM -1 | A tells B he is very happy. B picks something up | 0 | A sagt B dass er sehr glücklich ist. B hebt etwas auf |
|  | COM -2 | A asks B to pick something up. B picks something up | 0 | bittet B etwas aufzuheben. B hebt etwas auf |
|  | IND -1 | A moves over. B picks something up | 5 | A geht zur Seite. B hebt etwas auf |
|  | IND-2 | A lifts something. B picks something up | 0 | A hebt etwas. B hebt etwas auf |
| Lateral steps | **Com vs Ind %** |  | **85** |  |
|  | **Correct (IND)** | **A makes some lateral steps. B takes something and eats it** | **80** | **A macht ein paar seitliche Schritte. B nimmt etwas und isst es** |
|  | COM -1 | A offers something to B. B takes something and tastes it | 10 | A bietet B etwas an. B nimmt etwas und isst es |
|  | COM -2 | A asks B to move over. B takes something and eats it | 0 | A bittet B zur Seite zu gehen. B nimmt etwas und isst es |
|  | IND -1 | A turns around. B takes something and eats it | 5 | A dreht sich um. B nimmt etwas und isst es |
|  | IND-2 | A pushes something. B takes something and eats it | 5 | A schiebt etwas. B nimmt etwas und isst es |
| Look under the foot | **Com vs Ind %** |  | **90** |  |
|  | **Correct (IND)** | **A looks under his foot. B moves something** | **100** | **A schaut unter seinen Fuß. B bewegt etwas** |
|  | COM -1 | A asks B to move something. B moves something | 0 | A bittet B etwas zu bewegen. B bewegt etwas |
|  | COM -2 | A asks B to look at something. B moves something | 0 | A bittet B sich etwas anzuschauen. B bewegt etwas |
|  | IND -1 | A kicks something. B moves something | 0 | A tritt nach etwas. B bewegt etwas |
|  | IND-2 | A turns around. B moves something | 0 | A dreht sich um. B bewegt etwas |
| Sneeze | **Com vs Ind %** |  | **80** |  |
|  | **Correct (IND)** | **A sneezes. B turns around** | **80** | **A niest. B dreht sich um** |
|  | COM -1 | A asks B to look at something. B turns around | 5 | A bittet B sich etwas anzuschauen. B dreht sich um |
|  | COM -2 | A asks B to sit down. B turns around | 5 | A bittet B sich zu setzen. B dreht sich um |
|  | IND -1 | A eats something. B turns around | 0 | A isst etwas. B dreht sich um |
|  | IND-2 | A throws something on the ground. B turns around | 10 | A wirft etwas zu Boden. B dreht sich um |
| Stretch | **Com vs Ind %** |  | **85** |  |
|  | **Correct (IND)** | **A stretches. B moves something** | **75** | **A macht Dehnübungen. B bewegt etwas** |
|  | COM -1 | A asks B to move something. B moves something | 0 | A bittet B etwas zu bewegen. B bewegt etwas |
|  | COM -2 | A asks B to squat down. B moves something | 5 | A bittet B in die Hocke zu gehen. B bewegt etwas |
|  | IND -1 | A picks something up. B moves something | 15 | A hebt etwas auf. B bewegt etwas |
|  | IND-2 | A moves something. B moves something | 5 | A bewegt etwas. B bewegt etwas |
| Turn over | **Com vs Ind %** |  | **95** |  |
|  | **Correct (IND)** | **A turns over. B squats down** | **100** | **A dreht sich um. B bückt sich** |
|  | COM -1 | A shows something to B. squats down | 0 | A zeigt B etwas. B bückt sich |
|  | COM -2 | A asks B to move away. B squats down | 0 | A bittet B wegzugehen. B bückt sich |
|  | IND -1 | A moves something. B squats down | 0 | A bewegt etwas. B bückt sich |
|  | IND-2 | A moves away. B squats down | 0 | A geht weg. B bückt sich |
