# Supplementary material for: The Multilingual CID-5: A New Tool to Study the Perception of Communicative Interactions in Different Languages
Source: Front Psychol. 2015 Nov 17;6:1724. doi: 10.3389/fpsyg.2015.01724 (PMC4648072; doi:10.3389/fpsyg.2015.01724)
Supplement: Supplementary file 2 [file Data_Sheet_1.ZIP › Alternatives/italian.doc]

| **Action** | **Alternative** | **Alternatives** | **Italian**  **% (N=20)** |  |
| --- | --- | --- | --- | --- |
| Choose which one | **Com vs Ind %** |  | **95** |  |
|  | **Correct (COM)** | **A asks B to choose between two objects. B takes an object** | **55** | **A chiede a B di scegliere tra due oggetti. B prende un oggetto** |
|  | COM -1 | A offers something to B. B takes an object | 15 | A offre a B qualcosa. B prende un oggetto |
|  | COM -2 | A squats down and asks B to imitate him. B takes an object | 5 | A fa un piegamento e chiede a B di imitarlo. B prende un oggetto |
|  | IND -1 | A lifts something. B takes an object | 15 | A solleva un oggetto. B prende un oggetto |
|  | IND-2 | A weights something in his hands. B takes an object | 10 | A soppesa un oggetto. B prende un oggetto |
| Come closer | **Com vs Ind %** |  | **100** |  |
|  | **Correct (COM)** | **A asks B to come closer. B moves forward** | **70** | **A chiede a B di avvicinarsi. B fa qualche passo avanti** |
|  | COM -1 | A shows something to B. B moves forward | 0 | A mostra qualcosa a B. B fa qualche passo avanti |
|  | COM -2 | A waves to B. B moves forward | 30 | A saluta. B fa qualche passo avanti |
|  | IND -1 | A drinks. B moves forward | 0 | A beve. B fa qualche passo avanti |
|  | IND-2 | A stretches. B moves forward | 0 | A fa stretching. B fa qualche passo avanti |
| Go out of the way | **Com vs Ind %** |  | **100** |  |
|  | **Correct (COM)** | **A asks B to go out of the way. B moves over** | **85** | **A chiede a B di spostarsi. B si sposta** |
|  | COM -1 | A say hello to B. B moves over | 0 | A saluta B. B si sposta |
|  | COM -2 | A asks B to hand him something. B moves over | 10 | A chiede a B di dargli qualcosa. B si sposta |
|  | IND -1 | A scratches himself. B moves over | 0 | A si gratta. B si sposta |
|  | IND-2 | A cleans something. B moves over | 5 | A pulisce qualcosa. B si sposta |
| Imitate me | **Com vs Ind %** |  | **85** |  |
|  | **Correct (COM)** | **A squats down, and asks B to imitate him. B squats down** | **85** | **A fa un piegamento, e chiede a B di imiatarlo. B si piega** |
|  | COM -1 | A gives something to B. B squats down | 5 | A porge qualcosa a B. B si piega |
|  | COM -2 | A asks B to come closer. B squats down | 0 | A chiede a B di avvicinarsi. B si piega |
|  | IND -1 | A sits down. B squats down | 10 | A si siede. B si piega |
|  | IND-2 | A puts something down. B squats down | 0 | A appoggia qualcosa. B si piega |
| Look at the ceiling | **Com vs Ind %** |  | **65** |  |
|  | **Correct (COM)** | **A asks B to look at something behind him on the ceiling. B turns around** | **40** | **A chiede a B di guardare qualcosa in alto. B si gira** |
|  | COM -1 | A asks B to turn. B turns around | 10 | A chiede a B di girare su se stesso. B si gira |
|  | COM -2 | A asks B to come closer. B turns around | 15 | A chiede di avvicinarsi. B si gira |
|  | IND -1 | A drinks. B turns around | 25 | A beve. B si gira |
|  | IND-2 | A puts something down. B turns around | 10 | A solleva qualcosa. B si gira |
| Look at the ground | **Com vs Ind %** |  | **100** |  |
|  | **Correct (COM)** | **A asks B to look at something on the ground. B squats down** | **90** | **A chiede a B di guardare qualcosa per terra. B si abbassa** |
|  | COM -1 | A squats down and asks B to imitate him. B squats down | 5 | A fa un piegamento, e chiede a B di imitarlo. B si abbassa |
|  | COM -2 | A asks B to move away. B squats down | 0 | A chiede a B di allontanarsi. B si abbassa |
|  | IND -1 | A sits down. B squats down | 0 | A si siede. B si abbassa |
|  | IND-2 | A picks something up. B squats down | 5 | A raccoglie qualcosa. B si abbassa |
| Move this down | **Com vs Ind %** |  | **55** |  |
|  | **Correct (COM)** | **A asks B to move something. B moves something** | **55** | **A chiede a B di spostare qualcosa. B sposta qualcosa** |
|  | COM -1 | A asks B to imitate him. B moves something | 0 | A chiede a B di imitarlo. B sposta qualcosa |
|  | COM -2 | A asks B to squat down. B moves something | 0 | A chiede a B di abbassarsi. B sposta qualcosa |
|  | IND -1 | A pours something. B moves something | 40 | A si versa da bere. B sposta qualcosa |
|  | IND-2 | A moves something. B moves something | 5 | A sposta qualcosa. B sposta qualcosa |
| No | **Com vs Ind %** |  | **95** |  |
|  | **Correct (COM)** | **A says ‘No’. B stops** | **95** | **A dice 'No'. B si ferma** |
|  | COM -1 | A says ‘Hi’. B stops | 0 | A saluta. B si ferma |
|  | COM -2 | A asks B to get out of the way. B stops | 0 | A chiede a B di spostarsi. B si ferma |
|  | IND -1 | A rubs something. B stops | 5 | A cancella qualcosa. B si ferma |
|  | IND-2 | A shakes something. B stops | 0 | A scrolla qualcosa. B si ferma |
| Pick this up | **Com vs Ind %** |  | **85** |  |
|  | **Correct (COM)** | **A points to B something to pick up. B picks something up** | **75** | **A indica a B qualcosa da raccogliere. B raccoglie qualcosa** |
|  | COM -1 | A asks B to squat down. B picks something up | 10 | A chiede a B di abbassarsi. B raccoglie qualcosa |
|  | COM -2 | A asks B to stop. B picks something up | 0 | A chiede a B di fermarsi. B raccoglie qualcosa |
|  | IND -1 | A moves something. B picks something up | 15 | A sposta qualcosa. B raccoglie qualcosa |
|  | IND-2 | A checks the time. B picks something up | 0 | A guarda l'ora. B raccoglie qualcosa |
| Sit down | **Com vs Ind %** |  | **80** |  |
|  | **Correct (COM)** | **A asks B to sit down. B sits down** | **65** | **A chiede a B di sedersi. B si siede** |
|  | COM -1 | A asks B to move over. B sits down | 10 | A chiede a B di allontanarsi. B si siede |
|  | COM -2 | A gives something to B. B sits down | 5 | A porge qualcosa a B. B si siede |
|  | IND -1 | A puts something down. B sits down | 5 | A appoggia qualcosa. B si siede |
|  | IND-2 | A folds something. B sits down | 15 | A piega qualcosa. B si siede |
| Squat down | **Com vs Ind %** |  | **85** |  |
|  | **Correct (COM)** | **A asks B to squat down. B squats down** | **80** | **A chiede a B di abbassarsi. B si abbassa** |
|  | COM -1 | A asks B to pick something up. B squats down | 5 | A chiede a B di raccogliere qualcosa. B si abbassa |
|  | COM -2 | A asks B to walk away. B squats down | 0 | A chiede a B di allontanarsi. B si abbassa |
|  | IND -1 | A cleans something. B squats down | 10 | A pulisce qualcosa. B si abbassa |
|  | IND-2 | A bounces a ball. B squats down | 5 | A fa rimbalzare una palla. B si abbassa |
| Stand up | **Com vs Ind %** |  | **100** |  |
|  | **Correct (COM)** | **A asks B to stand up. B stands up** | **95** | **A chiede a B di alzarsi in piedi. B si alza in piedi** |
|  | COM -1 | A shows something to B. B stands up | 0 | A mostra qualcosa a B. B si alza in piedi |
|  | COM -2 | A asks B to squat down. B stands up | 0 | A chiede a B di abbassarsi. B si alza in piedi |
|  | IND -1 | A bounces a ball. B stands up | 0 | A fa rimbalzare una palla. B si alza in piedi |
|  | IND-2 | A paints something. B stands up | 5 | A tinteggia. B si alza in piedi |
| Stop | **Com vs Ind %** |  | **100** |  |
|  | **Correct (COM)** | **A asks B to stop. B stops** | **90** | **A chiede a B di fermarsi. B si ferma** |
|  | COM -1 | A shows something to B. B stops | 10 | A mostra qualcosa a B. B si ferma |
|  | COM -2 | A asks B to sit down. B stops | 0 | A chiede a B di sedersi. B si ferma |
|  | IND -1 | A drinks. B stops | 0 | A beve. B si ferma |
|  | IND-2 | A puts something down. B stops | 5 | A solleva qualcosa. B si ferma |
| Walk away | **Com vs Ind %** |  | **65** |  |
|  | **Correct (COM)** | **A asks B to walk away. B takes some steps** | **55** | **A chiede a B di anadare in quella direzione. B fa qualche passo** |
|  | COM -1 | A opens the door for B. B takes some steps | 30 | A apre la porta a B. B fa qualche passo |
|  | COM -2 | A asks B to move something. B takes some steps | 0 | A chiede a B di spostare qualcosa. B fa qualche passo |
|  | IND -1 | A stretches. B takes some steps | 5 | A fa stretching. B fa qualche passo |
|  | IND-2 | A draws a line. B takes some steps | 10 | A traccia una riga. B fa qualche passo |
| Drink | **Com vs Ind %** |  | **90** |  |
|  | **Correct (IND)** | **A drinks. B sits down** | **80** | **A beve. B si siede** |
|  | COM -1 | A asks B to sit down. B sits down | 10 | A chiede a B di sedersi. B si siede |
|  | COM -2 | A asks B to look at something. B sits down | 0 | A chiede a B di guardare qualcosa. B si siede |
|  | IND -1 | A looks at the time. B sits down | 5 | A guarda l'ora. B si siede |
|  | IND-2 | A scratches his head. B sits down | 5 | A si gratta. B si siede |
| Jump | **Com vs Ind %** |  | **90** |  |
|  | **Correct (IND)** | **A jumps. B picks something up** | **100** | **A salta. B raccoglie qualcosa** |
|  | COM -1 | A tells B he is very happy. B picks something up | 0 | A dice a B che è molto contento. B raccoglie qualcosa |
|  | COM -2 | A asks B to pick something up. B picks something up | 0 | A chiede a B di raccogliere qualcosa. B raccoglie qualcosa |
|  | IND -1 | A moves over. B picks something up | 0 | A si sposta. B raccoglie qualcosa |
|  | IND-2 | A lifts something. B picks something up | 0 | A solleva qualcosa. B raccoglie qualcosa |
| Lateral steps | **Com vs Ind %** |  | **90** |  |
|  | **Correct (IND)** | **A makes some lateral steps. B takes something and eats it** | **55** | **A fa dei passi laterali. B prende qualcosa e lo mangia** |
|  | COM -1 | A offers something to B. B takes something and tastes it | 15 | A offre qualcosa a B. B prende qualcosa e lo mangia |
|  | COM -2 | A asks B to move over. B takes something and eats it | 5 | A chiede a B di spostarsi. B prende qualcosa e lo mangia |
|  | IND -1 | A turns around. B takes something and eats it | 15 | A si gira. B prende qualcosa e lo mangia |
|  | IND-2 | A pushes something. B takes something and eats it | 10 | A spinge qualcosa. B prende qualcosa e lo mangia |
| Look under the foot | **Com vs Ind %** |  | **100** |  |
|  | **Correct (IND)** | **A looks under his foot. B moves something** | **100** | **A guarda sotto il suo piede. B sposta qualcosa** |
|  | COM -1 | A asks B to move something. B moves something | 0 | A chiede a B di spostare qualcosa. B sposta qualcosa |
|  | COM -2 | A asks B to look at something. B moves something | 0 | A chiede a B di guardare qualcosa. B sposta qualcosa |
|  | IND -1 | A kicks something. B moves something | 0 | A colpisce qualcosa. B sposta qualcosa |
|  | IND-2 | A turns around. B moves something | 0 | A gira su se stesso. B sposta qualcosa |
| Sneeze | **Com vs Ind %** |  | **100** |  |
|  | **Correct (IND)** | **A sneezes. B turns around** | **85** | **A starnutisce. B si gira** |
|  | COM -1 | A asks B to look at something. B turns around | 0 | A chiede a B di guardare qualcosa. B si gira |
|  | COM -2 | A asks B to sit down. B turns around | 5 | A chiede a B di sedersi. B si gira |
|  | IND -1 | A eats something. B turns around | 0 | A mangia qualcosa. B si gira |
|  | IND-2 | A throws something on the ground. B turns around | 10 | A getta qualcosa per terra. B si gira |
| Stretch | **Com vs Ind %** |  | **100** |  |
|  | **Correct (IND)** | **A stretches. B moves something** | **85** | **A fa stretching. B sposta qualcosa** |
|  | COM -1 | A asks B to move something. B moves something | 0 | A chiede a B di spostare qualcosa. B sposta qualcosa |
|  | COM -2 | A asks B to squat down. B moves something | 0 | A chiede a B di abbassarsi. B sposta qualcosa |
|  | IND -1 | A picks something up. B moves something | 15 | A raccoglie qualcosa. B sposta qualcosa |
|  | IND-2 | A moves something. B moves something | 0 | A sposta qualcosa. B sposta qualcosa |
| Turn over | **Com vs Ind %** |  | **85** |  |
|  | **Correct (IND)** | **A turns over. B squats down** | **75** | **A si gira. B si abbassa** |
|  | COM -1 | A shows something to B. squats down | 5 | A mostra qualcosa a B. B si abbassa |
|  | COM -2 | A asks B to move away. B squats down | 0 | A chiede a B di allontanarsi. B si abbassa |
|  | IND -1 | A moves something. B squats down | 5 | A sposta qualcosa. B si abbassa |
|  | IND-2 | A moves away. B squats down | 15 | A si allontana. B si abbassa |
