# Supplementary material for: The Multilingual CID-5: A New Tool to Study the Perception of Communicative Interactions in Different Languages
Source: Front Psychol. 2015 Nov 17;6:1724. doi: 10.3389/fpsyg.2015.01724 (PMC4648072; doi:10.3389/fpsyg.2015.01724)
Supplement: Supplementary file 2 [file Data_Sheet_1.ZIP › Alternatives/polish.doc]

| **Action** | **Alternative** | **Alternatives** | **Polish**  **% (N=20)** |  |
| --- | --- | --- | --- | --- |
| Choose which one | **Com vs Ind %** |  | **100** |  |
|  | **Correct (COM)** | **A asks B to choose between two objects. B takes an object** | **40** | **A poleca B by wybrał jeden z dwóch przedmiotów. B bierze coś do ręki.** |
|  | COM -1 | A offers something to B. B takes an object | 35 | A proponuje coś B. B bierze coś do ręki. |
|  | COM -2 | A squats down and asks B to imitate him. B takes an object | 0 | A kuca i poleca B by go naśladował. B bierze coś do ręki. |
|  | IND -1 | A lifts something. B takes an object | 15 | A podnosi coś. B bierze coś do ręki. |
|  | IND-2 | A weights something in his hands. B takes an object | 10 | A waży coś w ręku. B bierze coś do ręki. |
| Come closer | **Com vs Ind %** |  | **95** |  |
|  | **Correct (COM)** | **A asks B to come closer. B moves forward** | **75** | **A poleca B by podszedł bliżej. B podchodzi do A.** |
|  | COM -1 | A shows something to B. B moves forward | 5 | A pokazuje coś B. B podchodzi do A. |
|  | COM -2 | A waves to B. B moves forward | 20 | A wita się z B machając. B podchodzi do A. |
|  | IND -1 | A drinks. B moves forward | 0 | A pije. B podchodzi do A. |
|  | IND-2 | A stretches. B moves forward | 0 | A rozciąga się. B podchodzi do A. |
| Go out of the way | **Com vs Ind %** |  | **90** |  |
|  | **Correct (COM)** | **A asks B to go out of the way. B moves over** | **55** | **A poleca B stanąć z boku. B przesuwa się.** |
|  | COM -1 | A say hello to B. B moves over | 30 | A macha do B. B przesuwa się. |
|  | COM -2 | A asks B to hand him something. B moves over | 5 | A poleca B by coś mu podał. B przesuwa się. |
|  | IND -1 | A scratches himself. B moves over | 5 | A drapie się. B przesuwa się. |
|  | IND-2 | A cleans something. B moves over | 5 | A czyści coś. B przesuwa się. |
| Imitate me | **Com vs Ind %** |  | **95** |  |
|  | **Correct (COM)** | **A squats down, and asks B to imitate him. B squats down** | **95** | **A robi przysiad i poleca B by go naśladował. B robi przysiad.** |
|  | COM -1 | A gives something to B. B squats down | 0 | A daje coś B. B robi przysiad. |
|  | COM -2 | A asks B to come closer. B squats down | 0 | A poleca B by podszedł do niego. B robi przysiad. |
|  | IND -1 | A sits down. B squats down | 5 | A siada. B robi przysiad. |
|  | IND-2 | A puts something down. B squats down | 0 | A odkłada coś. B robi przysiad. |
| Look at the ceiling | **Com vs Ind %** |  | **85** |  |
|  | **Correct (COM)** | **A asks B to look at something behind him on the ceiling. B turns around** | **40** | **A poleca B by spojrzał na coś co wisi za nim na suficie. B obraca się.** |
|  | COM -1 | A asks B to turn. B turns around | 30 | A poleca B by się obrócił. B obraca się. |
|  | COM -2 | A asks B to come closer. B turns around | 20 | A poleca B by podszedł bliżej. B obraca się. |
|  | IND -1 | A drinks. B turns around | 0 | A pije. B obraca się. |
|  | IND-2 | A puts something down. B turns around | 10 | A odkłada coś. B obraca się. |
| Look at the ground | **Com vs Ind %** |  | **100** |  |
|  | **Correct (COM)** | **A asks B to look at something on the ground. B squats down** | **65** | **A zwraca uwagę B na coś leżącego na podłodze. B kuca.** |
|  | COM -1 | A squats down and asks B to imitate him. B squats down | 35 | A kuca i poleca B by go naśladował. B kuca. |
|  | COM -2 | A asks B to move away. B squats down | 0 | A poleca B by odszedł. B kuca. |
|  | IND -1 | A sits down. B squats down | 0 | A siada. B kuca. |
|  | IND-2 | A picks something up. B squats down | 0 | A podnosi coś. B kuca. |
| Move this down | **Com vs Ind %** |  | **90** |  |
|  | **Correct (COM)** | **A asks B to move something. B moves something** | **75** | **A poleca B by przestawił coś na ziemię. B przenosi coś.** |
|  | COM -1 | A asks B to imitate him. B moves something | 5 | A poleca B by go naśladował. B przenosi coś. |
|  | COM -2 | A asks B to squat down. B moves something | 0 | A poleca B by ukucnął. B przenosi coś. |
|  | IND -1 | A pours something. B moves something | 20 | A nalewa coś do szklanki. B przenosi coś. |
|  | IND-2 | A moves something. B moves something | 0 | A przenosi coś. B przenosi coś. |
| No | **Com vs Ind %** |  | **100** |  |
|  | **Correct (COM)** | **A says ‘No’. B stops** | **85** | **A mówi “Nie”. B zatrzymuje się.** |
|  | COM -1 | A says ‘Hi’. B stops | 10 | A mówi “Cześć”. B zatrzymuje się. |
|  | COM -2 | A asks B to get out of the way. B stops | 0 | A poleca B by zszedł mu z drogi. B zatrzymuje się. |
|  | IND -1 | A rubs something. B stops | 0 | A coś wyciera. B zatrzymuje się. |
|  | IND-2 | A shakes something. B stops | 5 | A potrząsa czymś. B zatrzymuje się. |
| Pick this up | **Com vs Ind %** |  | **95** |  |
|  | **Correct (COM)** | **A points to B something to pick up. B picks something up** | **90** | **A wskazuje na coś, co B ma podnieść. B podnosi coś.** |
|  | COM -1 | A asks B to squat down. B picks something up | 0 | A poleca B by ukucnął. B podnosi coś. |
|  | COM -2 | A asks B to stop. B picks something up | 0 | A poleca B by się zatrzymał. B podnosi coś. |
|  | IND -1 | A moves something. B picks something up | 0 | A przesuwa coś. B podnosi coś. |
|  | IND-2 | A checks the time. B picks something up | 10 | A patrzy na zegarek. B podnosi coś. |
| Sit down | **Com vs Ind %** |  | **95** |  |
|  | **Correct (COM)** | **A asks B to sit down. B sits down** | **95** | **A poleca B by usiadł. B siada.** |
|  | COM -1 | A asks B to move over. B sits down | 0 | A poleca B by przesunął się. B siada. |
|  | COM -2 | A gives something to B. B sits down | 0 | A podaje coś B. B siada. |
|  | IND -1 | A puts something down. B sits down | 5 | A odkłada coś. B siada. |
|  | IND-2 | A folds something. B sits down | 0 | A składa coś. B siada. |
| Squat down | **Com vs Ind %** |  | **100** |  |
|  | **Correct (COM)** | **A asks B to squat down. B squats down** | **85** | **A poleca B by ukucnął. B kuca.** |
|  | COM -1 | A asks B to pick something up. B squats down | 10 | A poleca B by coś podniósł. B kuca. |
|  | COM -2 | A asks B to walk away. B squats down | 0 | A poleca B by odszedł. B kuca. |
|  | IND -1 | A cleans something. B squats down | 0 | A czyści coś. B kuca. |
|  | IND-2 | A bounces a ball. B squats down | 5 | A odbija piłkę. B kuca. |
| Stand up | **Com vs Ind %** |  | **100** |  |
|  | **Correct (COM)** | **A asks B to stand up. B stands up** | **75** | **A poleca B by wstał. B wstaje.** |
|  | COM -1 | A shows something to B. B stands up | 20 | A pokazuje coś B. B wstaje. |
|  | COM -2 | A asks B to squat down. B stands up | 0 | A poleca B by ukucnął. B wstaje. |
|  | IND -1 | A bounces a ball. B stands up | 5 | A odbija piłkę. B wstaje. |
|  | IND-2 | A paints something. B stands up | 0 | A maluje coś. B wstaje. |
| Stop | **Com vs Ind %** |  | **100** |  |
|  | **Correct (COM)** | **A asks B to stop. B stops** | **95** | **A poleca B by się zatrzymał. B zatrzymuje się.** |
|  | COM -1 | A shows something to B. B stops | 5 | A pokazuje coś B. B zatrzymuje się. |
|  | COM -2 | A asks B to sit down. B stops | 0 | A poleca B by usiadł. B zatrzymuje się. |
|  | IND -1 | A drinks. B stops | 0 | A pije coś. B zatrzymuje się. |
|  | IND-2 | A puts something down. B stops | 0 | A odkłada coś. B zatrzymuje się. |
| Walk away | **Com vs Ind %** |  | **90** |  |
|  | **Correct (COM)** | **A asks B to walk away. B takes some steps** | **55** | **A poleca B by przeszedł. B robi kilka kroków.** |
|  | COM -1 | A opens the door for B. B takes some steps | 35 | A otwiera drzwi dla B. B robi kilka kroków. |
|  | COM -2 | A asks B to move something. B takes some steps | 0 | A poleca B by coś przesunął. B robi kilka kroków. |
|  | IND -1 | A stretches. B takes some steps | 0 | A rozciąga się. B robi kilka kroków. |
|  | IND-2 | A draws a line. B takes some steps | 10 | A rysuje linię. B robi kilka kroków. |
| Drink | **Com vs Ind %** |  | **80** |  |
|  | **Correct (IND)** | **A drinks. B sits down** | **75** | **A pije coś. B siada.** |
|  | COM -1 | A asks B to sit down. B sits down | 10 | A poleca B by usiadł. B siada. |
|  | COM -2 | A asks B to look at something. B sits down | 10 | A poleca B by spojrzał na coś. B siada. |
|  | IND -1 | A looks at the time. B sits down | 0 | A patrzy na zegarek. B siada. |
|  | IND-2 | A scratches his head. B sits down | 5 | A drapie się po głowie. B siada. |
| Jump | **Com vs Ind %** |  | **90** |  |
|  | **Correct (IND)** | **A jumps. B picks something up** | **85** | **A podskakuje. B podnosi coś z ziemi.** |
|  | COM -1 | A tells B he is very happy. B picks something up | 5 | A mówi B, że się cieszy. B podnosi coś z ziemi. |
|  | COM -2 | A asks B to pick something up. B picks something up | 10 | A poleca B by coś podniósł. B podnosi coś z ziemi. |
|  | IND -1 | A moves over. B picks something up | 0 | A przesuwa się. B podnosi coś z ziemi. |
|  | IND-2 | A lifts something. B picks something up | 0 | A podnosi coś. B podnosi coś z ziemi. |
| Lateral steps | **Com vs Ind %** |  | **85** |  |
|  | **Correct (IND)** | **A makes some lateral steps. B takes something and eats it** | **60** | **A robi krok w bok. B bierze coś i zjada to.** |
|  | COM -1 | A offers something to B. B takes something and tastes it | 25 | A oferuje coś B. B bierze coś i zjada to. |
|  | COM -2 | A asks B to move over. B takes something and eats it | 0 | A poleca B by odsunął się. B bierze coś i zjada to. |
|  | IND -1 | A turns around. B takes something and eats it | 0 | A obraca się. B bierze coś i zjada to. |
|  | IND-2 | A pushes something. B takes something and eats it | 15 | A popycha coś. B bierze coś i zjada to. |
| Look under the foot | **Com vs Ind %** |  | **80** |  |
|  | **Correct (IND)** | **A looks under his foot. B moves something** | **90** | **A ogląda spód swojej stopy. B przenosi coś.** |
|  | COM -1 | A asks B to move something. B moves something | 0 | A poleca B by coś przeniósł. B przenosi coś. |
|  | COM -2 | A asks B to look at something. B moves something | 5 | A poleca B by spojrzał na coś. B przenosi coś. |
|  | IND -1 | A kicks something. B moves something | 5 | A kopie w coś. B przenosi coś. |
|  | IND-2 | A turns around. B moves something | 0 | A obraca się. B przenosi coś. |
| Sneeze | **Com vs Ind %** |  | **50** |  |
|  | **Correct (IND)** | **A sneezes. B turns around** | **75** | **A kicha. B obraca się.** |
|  | COM -1 | A asks B to look at something. B turns around | 15 | A poleca B by spojrzał na coś. B obraca się. |
|  | COM -2 | A asks B to sit down. B turns around | 5 | A poleca B by usiadł. B obraca się. |
|  | IND -1 | A eats something. B turns around | 0 | A je coś. B obraca się. |
|  | IND-2 | A throws something on the ground. B turns around | 5 | A rzuca coś na ziemię. B obraca się. |
| Stretch | **Com vs Ind %** |  | **70** |  |
|  | **Correct (IND)** | **A stretches. B moves something** | **45** | **A rozciąga się. B przenosi coś.** |
|  | COM -1 | A asks B to move something. B moves something | 5 | A poleca B by coś przesunął. B przenosi coś. |
|  | COM -2 | A asks B to squat down. B moves something | 0 | A poleca B by ukucnął. B przenosi coś. |
|  | IND -1 | A picks something up. B moves something | 40 | A podnosi coś z ziemi. B przenosi coś. |
|  | IND-2 | A moves something. B moves something | 10 | A przenosi coś. B przenosi coś. |
| Turn over | **Com vs Ind %** |  | **80** |  |
|  | **Correct (IND)** | **A turns over. B squats down** | **90** | **A obraca się. B schyla się.** |
|  | COM -1 | A shows something to B. squats down | 5 | A pokazuje coś B. B schyla się. |
|  | COM -2 | A asks B to move away. B squats down | 5 | A poleca B by się odsunął. B schyla się. |
|  | IND -1 | A moves something. B squats down | 0 | A przesuwa coś. B schyla się. |
|  | IND-2 | A moves away. B squats down | 0 | A odsuwa się. B schyla się. |
